# Substituent effect on the energy barrier for σ-bond formation from π-single-bonded species, singlet 2,2-dialkoxycyclopentane-1,3-diyls

**DOI:** 10.3762/bjoc.9.106

**Published:** 2013-05-14

**Authors:** Jianhuai Ye, Yoshihisa Fujiwara, Manabu Abe

**Affiliations:** 1 Department of Chemistry, Graduate School of Science, Hiroshima University, Japan; 2 Department of Mathematical and Life Sciences, Graduate School of Science, Hiroshima University, Japan,; 3 Institute for Molecular Science (IMS), Okazaki, Aichi 444-8787, Japan

**Keywords:** laser flash photolysis, lifetime, singlet diradicals (biradicals), substituent effect, π-single bond

## Abstract

**Background:** Localized singlet diradicals are in general quite short-lived intermediates in processes involving homolytic bond-cleavage and formation reactions. In the past decade, long-lived singlet diradicals have been reported in cyclic systems such as cyclobutane-1,3-diyls and cyclopentane-1,3-diyls. Experimental investigation of the chemistry of singlet diradicals has become possible. The present study explores the substituents and the effect of their substitution pattern at the C(1)–C(3) positions on the lifetime of singlet octahydropentalene-1,3-diyls to understand the role of the substituents on the reactivity of the localized singlet diradicals.

**Results:** A series of singlet 2,2-dialkoxy-1,3-diaryloctahydropentalene-1,3-diyls **DR** were generated in the photochemical denitrogenation of the corresponding azoalkanes **AZ**. The ring-closed products **CP**, i.e., 3,3-dialkoxy-2,4-diphenyltricyclo[3.3.0.0^2,4^]octanes, were quantitatively obtained in the denitrogenation reaction. The first-order decay process (*k* = 1/τ) was observed for the fate of the singlet diradicals **DR** (λ_max_ ≈ 580–590 nm). The activation parameters, Δ*H*^‡^ and Δ*S*^‡^, for the ring-closing reaction (σ-bond formation process) were determined by the temperature-dependent change of the lifetime. The energy barrier was found to be largely dependent upon the substituents Ar and Ar’. The singlet diradical **DRf** (Ar = 3,5-dimethoxyphenyl, OCH_2_Ar’ = OCH_2_(3,5-dimethoxyphenyl)) was the longest-lived, τ_293_ = 5394 ± 59 ns, among the diradicals studied here. The lifetime of the parent diradical **DR** (Ar = Ph, OCH_2_Ar’ = OCH_3_) was 299 ± 2 ns at 293 K.

**Conclusion:** The lifetimes of the singlet 1,3-diyls are found to be largely dependent on the substituent pattern of Ar and Ar’ at the C(1)–C(3) positions. Both the enthalpy and entropy effect were found to play crucial roles in increasing the lifetime.

## Introduction

**Figure 1:**
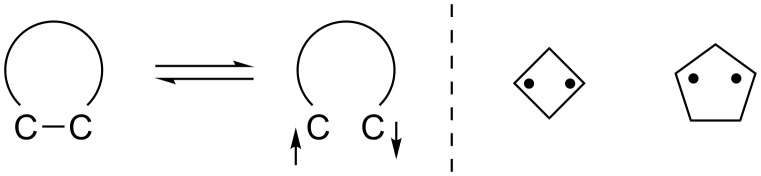
Localized singlet diradicals.

Localized singlet diradicals are key intermediates in processes involving the homolytic bond-cleavage and formation reactions (Figure 1) [1–2]. The singlet diradicals are, in general, quite short-lived species due to the very fast radical–radical coupling reaction [3]. However, in the past decade, the singlet diradicals have been observed or even isolated in cyclic systems such as cyclobutane-1,3-diyls [4–20] and cyclopentane-1,3-diyls [17,21–26]. Detailed experimental study of singlet diradical chemistry is thus now possible using the long-lived localized singlet diradicals.

**Scheme 1:**
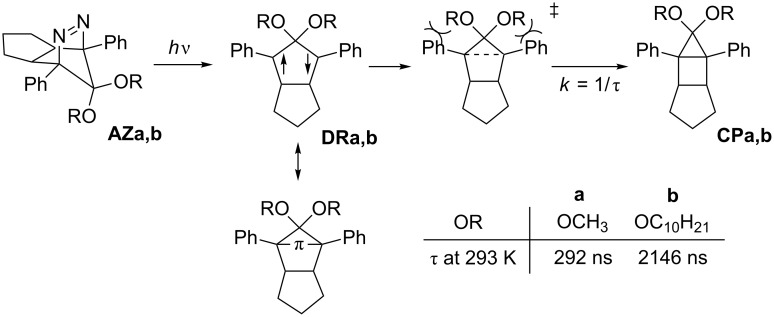
Alkoxy group effect on the lifetime of π-single-bonded species **DR**.

So far, we have studied singlet diradical chemistry using long-lived 2,2-dialkoxy-1,3-diphenyloctahydropentalene-1,3-diyls **DR** with a singlet ground state, which can be cleanly generated by the photochemical denitrogenation of the corresponding azoalkanes **AZ** (Scheme 1). The 2,2-electron-withdrawing-group-substituted singlet 1,3-diradicals are categorized as Type-1 diradicals [1,27], which possess a π-single-bonding character (–π–, closed-shell character) between the two radical sites. The role of the alkoxy group (OR) on the lifetime (*k* = 1/τ) was investigated by combined studies of experiments and quantum chemical calculations [26,28]. The steric repulsion between the alkoxy group and the phenyl ring, which is indicated in the transition-state structure for the ring-closing reaction (Scheme 1), was found to play an important role in determining the energy barrier of the ring-closing process, τ_293_ = 292 ns (**DRa**: OR = OCH_3_, λ_max_ = 574 nm) and 2146 ns (**DRb**: OR = OC_10_H_21_, λ_max_ = 572 nm) [26]. The study prompted us to further investigate the kinetic stabilization of the singlet diradical species.

**Scheme 2:**
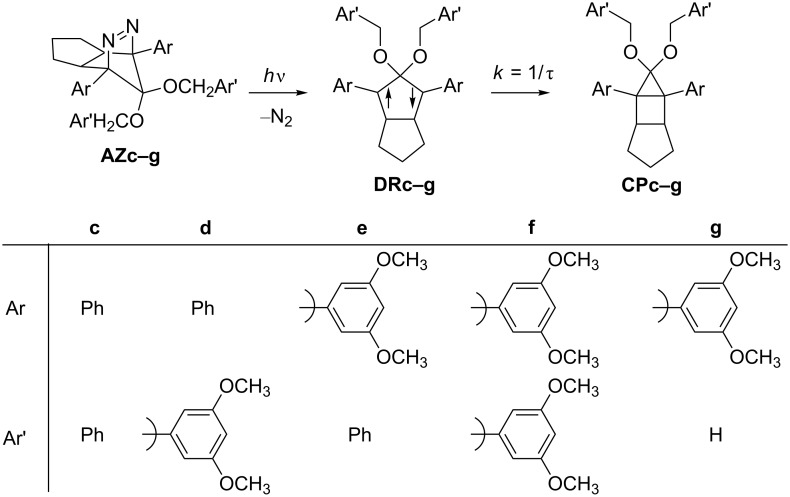
Generation of singlet diradicals **DRc**–**g** and their reactivity in the photochemical denitrogenation of **AZc**–**g**.

In the present study, the effect of the bulky 3,5-dimethoxyphenyl group substituent was investigated on the lifetime of the localized singlet diradicals. Thus, the aryl substituent was introduced at C(1), C(2), or/and C(3) positions of the diradicals **DRd**–**g**, and the substituent effects on the lifetime of the singlet diradicals were compared with the lifetime of a phenyl-group-substituted diradical **DRc** and the parent diradical **DRa**. The laser flash photolysis technique was used for the generation of **DRc**–**g** from the corresponding azoalkanes **AZc**–**g** (Scheme 2).

## Results and Discussion

**Scheme 3:**
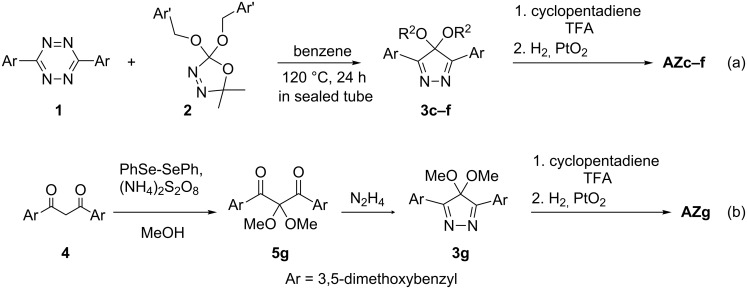
Synthesis of azoalkanes **AZc**–**f** and **AZg**.

**Synthesis of AZc–g and their steady-state photolyses.** The precursor azoalkanes **AZc**–**g** were prepared in an analogous method to the synthesis of **AZa,b** [28] (Scheme 3). Pyrazoles **3c**–**f** were synthesized in the reaction of tetrazines **1** (Ar = Ph or 3,5-dimethoxyphenyl) with 2,2-dialkoxy-5,5-dimethyl-Δ^3^-1,3,4-oxadiazolines **2** [29], which are the precursor of the dialkoxycarbene (Scheme 3a). Azoalkanes **AZc–f** (λ_max_ ≈ 360 nm with ε ≈ 100) were obtained by a cycloaddition reaction with cyclopentadienes, and followed by a hydrogenation reaction [30–31]. The synthesis of **AZg** (Ar = 3,5-dimethoxyphenyl, Ar’ = H) was performed from the corresponding 1,3-diketone **4** (Scheme 3b). 2,2-Dimethoxy-1,3-diarylpropane-1,3-dione **5g** was prepared from 1,3-dione **4** (R = 3,5-dimethoxybenzene) according to the method of Tiecco [32]. Pyrazole **3g** was then synthesized by the reaction with hydrazine hydrate. **AZg** was obtained by the Diels–Alder [4 + 2]-cycloaddition with cyclopentadiene and hydrogenation using PtO_2_ as a catalyst. The endo-configured structure of azoalkanes **AZc–g** was determined by X-ray crystallographic analysis as well as by NOE measurements.

The steady-state photolyses of **AZc–g** in benzene solution were performed with a Xenon lamp (500 W) through a Pyrex filter (*h*ν > 300 nm). The ring-closed compounds **CPc–g** were quantitatively obtained in the denitrogenation reaction (Scheme 2). The quantum yields of the denitrogenation of **AZc–g** were determined to be ≈0.90 by comparison with those reported for similar azoalkanes [33]. The quantitative formation of **CPc**–**g** and the high quantum yield of the denitrogenation process suggest the clean generation of **DRc**–**g** in the photoirradiation reaction of **AZc**–**g**.

**Figure 2:**
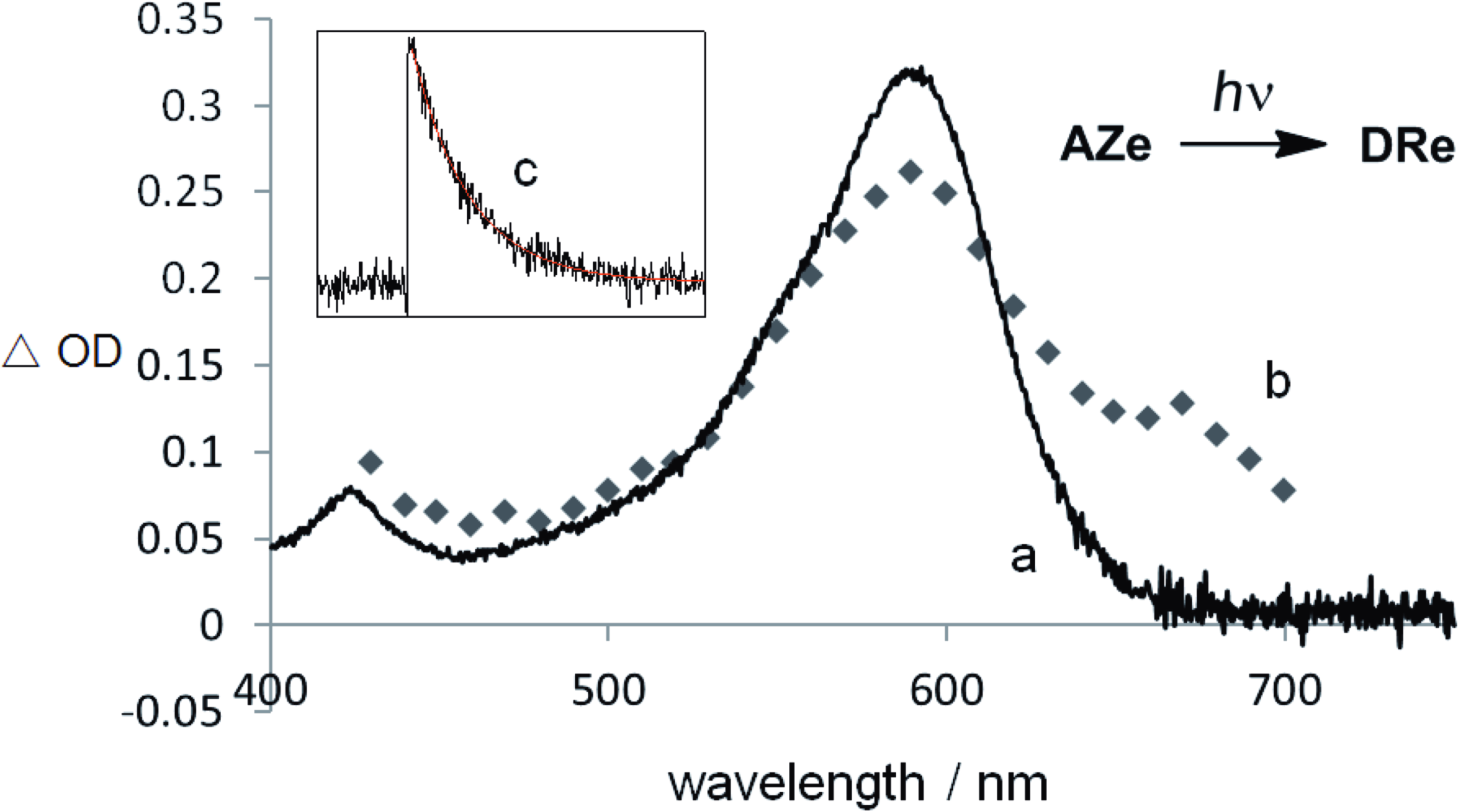
(a) Absorption spectrum of the singlet diradical **DRe** in a MTHF matrix at 80 K; (b) transient absorption spectrum of **AZe** measured immediately after the laser pulse (λ_exc_ = 355 nm); (c) transient decay trace at 580 nm and 20 °C.

**Detection of singlet diradicals DRc–g.** The detection of singlet diradicals **DRc**–**g** was examined by the photochemical denitrogenation of azoalkanes **AZc–g** in a glassy matrix of 2-methyltetrahydrofurane (MTHF) at 80 K, [**AZ**] ≈ 4 × 10^−3^ mol/L, and by the laser flash photolysis experiments of **AZc**–**g** at room temperature in benzene solution. First of all, the MTHF matrix solution of **AZ** was irradiated with a 500 W Xenon lamp through a monochromator (λ_irr_ = 360 ± 10 nm). A strong absorption band, which corresponds to **DRc–g**, was observed in the visible region at 80 K (570–590 nm, Table 1), as exemplified for the photoirradiation of **AZe** in Figure 2a. The strong absorption bands are quite similar to those of singlet diradicals **DRa,b** with λ_max_ = 574 nm and 572 nm [1,28], respectively. The assignment of the strong band to the singlet diradical is further supported by the following facts: (a) The absorptions obtained on photolysis in a MTHF glass were thermally persistent at 80 K and resembled that of the transient absorption spectra in solution (for example, **DRe**, λ_max_ = 590 nm, Figure 2b); (b) the species were ESR-silent in the MTHF-matrix at 80 K; (c) the lifetime of the transient was insensitive to the presence of molecular oxygen (decay trace at 580 nm, Figure 2c); and (d) the activation parameters (Table 1) are similar to those for the decay process of **DRa**, in particular, the high (ca. 10^12^ s^−1^) pre-exponential Arrhenius factors (log*A*) are indicative of a spin-allowed reaction to the ring-closed products **CPc–g** [34].

**Lifetime of singlet diradicals DRc–g and activation parameters for the ring-closing reaction**. The decay traces of the intermediary singlet diradicals **DRc–g** at 293–333 K were measured in a benzene solution by the laser flash photolysis technique (λ_exc_ = 355 nm). The lifetime (τ = 1/*k*) was determined by the first-order decay rate constants (*k*) of **DRc**–**g** at 580 nm, e.g., Figure 2c for **DRe**. As shown in Table 1, the lifetime of the singlet diradical was largely dependent on the substituents Ar and Ar’. The activation parameters, Δ*H*^‡^, Δ*S*^‡^, *E*_a_, log*A*, were determined from the Eyring plots and Arrhenius plots, which were obtained from the temperature-dependent change of the lifetime (Table 1). For comparison, the lifetime of diradical **DRa** (Table 1, entry 1) was also measured under similar conditions, and determined to be 299 ns at 293 K. The obtained lifetime was nearly the same as that obtained previously by us (292 ns) [28].

**Table 1:** Lifetimes and activation parameters of singlet diradicals **DR**.

entry	**DR**	τ_293K_ /ns^a^	λ_max_ /nm^b^ (at 80 K)	Δ*G*^‡^_293K_^c^ /kJ mol^−1^	Δ*H*^‡c^ /kJ mol^−1^	Δ*S*^‡c^ /J mol^−1^ K^−1^	*E*_a_^c^ /kJ mol^−1^	log *A*^c^

1	**DRa**	299	573	35.1 ± 0.7	32.7 ± 0.2	–8.1 ± 1.2	35.3 ± 0.2	12.8 ± 0.1
2	**DRc**	1305	583	39.1 ± 0.9	33.5 ± 0.6	–17.9 ± 1.7	36.2 ± 0.6	12.3 ± 0.1
3	**DRd**	1933	584	39.6 ± 0.6	36.6 ± 0.1	–10.1 ± 1.1	39.2 ± 0.1	12.7 ± 0.1
4	**DRe**	4001	592	40.9 ± 0.8	33.3 ± 0.4	–27.8 ± 1.3	35.9 ± 0.4	11.8 ± 0.1
5	**DRf**	5394	593	42.2 ± 0.7	36.5 ± 0.3	–19.4 ± 1.0	39.1 ± 0.3	12.2 ± 0.1
6	**DRg**	580	583	36.7 ± 0.4	33.0 ± 0.2	–12.9 ± 1.0	35.6 ± 0.2	12.2 ± 0.1

^a^Experimental errors are ca. 5%. ^b^In MTHF at 80 K. ^c^Activation parameters were determined by measurements of the lifetime of the singlet diradicals at five different temperatures in a temperature range from 293 to 333 K.

The lifetime of **DRc** (Ar = Ar’ = Ph) was found to be 1305 ns at 293 K (Table 1, entry 2), which was ca. 4.5 times longer than the parent **DRa**. On introduction of a 3,5-dimethoxyphenyl ring at C(2) position of the 1,3-diradical, i.e., **DRd** (Ar = Ph, Ar’ = 3,5-dimethoxyphenyl), a further increase of the lifetime at 293 K was observed to be 1933 ns (Table 1, entry 3). The result clearly indicates that the steric bulkiness plays an important role in increasing the energy barrier for the ring-closing reaction. Indeed, the activation enthalpy (Δ*H*^‡^ = 36.6 kJ mol^−1^, Table 1, entry 3) for **DRd** was found to be higher than that for **DRa** (Δ*H*^‡^ = 32.7 kJ mol^−1^, Table 1, entry 1). Interestingly, the effect of an aryl group substituent at C(1) and C(3) positions on the lifetime was found to be larger than that at C(2); compare the lifetime of **DRe** (4001 ns, Ar = 3,5-dimethoxyphenyl, Ar’ = Ph, Table 1, entry 4) with that of **DRd** (1933 ns, Table 1, entry 3). When the 3,5-dimethoxyphenyl group was introduced at all of the C(1), C(2), and C(3) positions, the lifetime of the diradical **DRf** (Δ*G*^‡^ = 42.2 kJ mol^−1^, Table 1, entry 5) was dramatically increased to 5394 ns at 293 K. The activation entropy (Δ*S*^‡^ = −27.8 and −19.4 J mol^−1^, Table 1, entries 4 and 5) also plays an important role in increasing the lifetime of the singlet species. A much shorter lifetime was found for the diradical **DRg** (Ar = 3,5-dimethoxyphenyl, Ar’ = H). Thus, the introduction of the bulky substituents is needed at all positions C(1)–C(3) of the 1,3-diradicals to increase the lifetime. The repulsive steric interactions of the Ar group with the Ar’ group are suggested to play important roles in increasing the energy barrier of the reaction from the diradicals to the ring-closed compounds **CP**. The results clearly indicate that the substituent effect using the sterically bulky group is effective to prolong the lifetime of the singlet diradicals.

## Conclusion

We have succeeded in generating long-lived singlet diradical species **DRc**–**g**, τ_293_ = 580–5394 ns, which were much longer-lived species than **DRa** (τ_293_ = 299 ns). It was found that the lifetimes are largely dependent on the substituent pattern of Ar and Ar’ at the C(1)–C(3) positions of the 1,3-diyls. Thus, both the enthalpy and entropy effect were found to play crucial roles in increasing the lifetime.

## Experimental

All reagents were purchased from commercial sources and were used without additional purification, unless otherwise mentioned. Azoalkanes **AZc–g** were prepared according to the methods described previously (Scheme 3) and were isolated by silica gel column chromatography and GPC column chromatography. ^1^H and ^13^C NMR spectra were reported in parts per million (δ) by using CDCl_3_ or C_6_D_6_ as internal standards. Assignments of ^13^C NMR were carried out by DEPT measurements. IR spectra were recorded with a FTIR spectrometer. UV–vis spectra were taken by a JASCO V-630 spectrophotometer. Mass-spectrometric data were measured by a Mass Spectrometric Thermo Fisher Scientific LTQ Orbitrap XL, performed by the Natural Science Center for Basic Research and Development (NBARD), Hiroshima University.

### Preparation of diazenes **AZc–g**

**3,6-Diaryl-1,2,4,5-tetrazine 1.** 3,6-Diphenyl-1,2,4,5-tetrazine was purchased and directly used. The preparation of 3,6-(3,5-dimethoxyphenyl)-1,2,4,5-tetrazine (Ar = 3,5-dimethoxyphenyl) is as follows: In a 50 mL round-bottom flask, benzonitrile (3.7 g, 22.7 mmol) was dissolved in 10 mL of absolute ethanol. Hydrazine (3.6 mL, 90 mmol) and sulfur (0.43 g, 13.5 mmol) were quickly added, and the solution was stirred at room temperature for 1 h and then heated under reflux for 3 h. The remaining orange cake was solidified further in an ice bath. The solid was vacuum filtered, and washed with cold ethanol (3 × 10 mL) giving the crude dihydrotetrazine. The crude orange solid was then placed in a 50 mL beaker and dissolved in 20% acetic acid (15 mL) and 10 mL ether at room temperature with stirring. An aqueous solution of 10% NaNO_2_ (20 mL) was added to the solution in an ice bath. The immediate purple cloudiness signifies the completion of the reaction, as well as the evolution of brown nitric oxide gas. Vacuum filtration and washing with hot methanol (3 × 10 mL) gave the tetrazine as a red solid (3.07 g, 81%). mp: 248–250 °C; IR (neat, cm^−1^): 3022, 2981, 2947, 1611, 1462, 1393, 1221, 1067, 944, 843, 683; ^1^H NMR (400 MHz, CDCl_3_) δ 3.88 (s, 6H), 6.60 (t, *J* = 2.23 Hz, 1H), 7.17 (d, *J* = 2.23 Hz, 2H); ^13^C NMR (125 MHz, CDCl_3_) δ 55.68 (q, OCH_3_), 105.48 (d, CH), 105.90 (d, CH), 133.44 (s, C), 161.54 (s, COCH_3_); ESIMS (*m*/*z*): [M + Na]^+^ calcd for C_18_H_20_O_4_N_4_, 379.13768; found, 379.13776.

**5,5-Dimethyl-2,2-bis(3,5-dimethoxybenzyl)-Δ^3^-1,3,4-oxadiazoline (2f).** A solution of (3,5-dimethoxybenzyloxycarbonyl)hydrazone of acetone (1.30 g, 4.87 mmol) in CH_2_Cl_2_ (5 mL) was added dropwise to a stirred solution of Pb(OAc)_4_ (2.59 g, 5.84 mmol) under nitrogen. The reaction mixture was stirred in an ice bath for 2 h, and then at room temperature for 24 h. After the stirring, the solid was filtered over Celite and the organic layer was washed with 10% aq NaHCO_3_. The mixture was filtered again until no precipitate was deposited. The organic phase was concentrated under reduced pressure. The corresponding 3,5-dimethoxybenzyl alcohol (2.46 g, 14.6 mmol) and TFA (0.04 mL, 0.49 mmol) were then added to the organic mixture. The solution was heated to 40 °C and stirred for 24 h before KOH pellets were added, and stirring was continued for another 3 h. After extracting with CH_2_Cl_2_, washing with brine, and drying by MgSO_4_, the organic layer was concentrated under reduced pressure. The product was purified by column chromatography (eluent: EtOAc/hexane = 30/70, *R*_f_ = 0.17) to give the product as a yellow liquid (0.48 g, 23%). IR (neat, cm^−1^): 3006, 2959, 2843, 1750, 1622, 1480, 1386, 1236, 1076, 925, 846, 687; ^1^H NMR (500 MHz, CDCl_3_) δ 1.49 (s, 6H), 3.67 (s, 12H), 4.66 (q, *J* = 11.8 Hz, 36.78 Hz, 4H), 6.29 (t, *J* = 2.21 Hz, 2H), 6.41 (d, *J* = 2.21 Hz, 4H); ^13^C NMR (125 MHz, CDCl_3_) δ 24.12 (q, CH_3_), 55.29 (q, OCH_3_), 66.72 (t, CH_2_), 99.94 (d, CH, Ar ring), 105.50 (d, CH, Ar ring), 119.63 (s, C(CH_3_)_2_), 136.71 (s, C), 138.97 (s, C(OCH_2_Ar)_2_), 160.83 (s, COCH_3_); ESIMS (*m*/*z*): [M + Na]^+^ calcd for C_22_H_28_O_7_N_2_Na, 455.17887; found, 455.17899.

**1,3-Bis(3,5-dimethoxyphenyl)propane-1,3-dione (4).** 3,5-dimethoxyacetophenone (2.1 g, 11.7 mmol), 3,5-dimethoxybenzoic acid (2.75 g, 14.04 mmol), and NaH (0.94 g, 23.4 mmol) was dissolved in THF (20 mL) under N_2_ atmosphere in a 50 mL flask. The mixture was heated under reflux (75 °C) for 14 h under a N_2_ atmosphere, then cooled down to room temperature. The mixture was slowly added to cold HCl. The organic layer was extracted with ether, washed with brine, and dried with anhydrous MgSO_4_. The solvent was removed by vacuum evaporator. The dry solid was then recrystallized in methanol to give the compound as a yellow crystal (2.77 g, 69%). mp: 132 °C; IR (neat, cm^−1^): 3140, 3000, 2943, 1562, 1466, 1351, 1298, 1158, 1053, 842, 668; ^1^H NMR (500 MHz, CDCl_3_) δ 3.78 (s, 12H), 6.56 (t, *J* = 2.28 Hz, 2H), 6.65 (d, *J* = 2.28 Hz, 4H); ^13^C NMR (125 MHz, CDCl_3_) δ 55.56 (q, OCH_3_), 93.56 (t, CH_2_), 104.62 (d, CH), 105.05 (d, CH), 137.52 (s, C), 160.92 (s, COCH_3_), 185.47 (s, OC=O); ESIMS (*m*/*z*): [M + Na]^+^ calcd for C_19_H_20_O_6_Na, 367.11521; found, 367.11456.

**1,3-Bis(3,5-dimethoxyphenyl)-2,2-dimethoxypropane-1,3-dione (5).** 1,3-Dione **4** (2.76 g, 8 mmol) and diphenyl diselenide (1.25 g, 4 mmol) were dissolved in methanol (50 mL), and ammonium persulfate (3.65 g, 16 mmol) was added to the mixture. The solution was heated under reflux for 4 h with stirring under nitrogen. Then the mixture was cooled to room temperature, and slowly added to ice water. The organic compound was extracted by chloroform and purified by silica-gel column chromatography (eluent: EtOAc/hexane = 30/70, *R*_f_ = 0.30) to give the product as a yellow liquid (2.9 g, 90%). ^1^H NMR (500 MHz, CDCl_3_) δ 3.41 (s, 6H), 3.77 (s, 12H), 6.59 (t, *J* = 2.44 Hz, 2H), 7.29 (d, *J* = 2.44 Hz, 4H); ^13^C NMR (125 MHz, CDCl_3_) δ 50.97 (q, C(OCH_3_)_2_), 55.53 (q, OCH_3_), 103.89 (s, C), 107.22 (d, CH), 107.34 (d, CH), 135.50 (s, C), 160.66 (s, COCH_3_), 192.47 (s, C=O); IR (neat, cm^−1^): 3027, 2965, 2845, 1691, 1605, 1466, 1429, 1321, 1162, 1070, 1036, 863, 671; ESIMS (*m*/*z*): [M + Na]^+^ calcd for C_21_H_24_O_8_Na, 427.13634; found, 427.13602.

#### 4,4-diaryloxy-3,5-diarylpyrazole (**3c–f**)

**General Procedure.** Oxadiazoline (1 mmol) was dissolved in benzene (2 mL) in a sealed tube. The mixture was stirred with tetrazine (1.10 mmol) in a sealed tube for 24 h at 120 °C under nitrogen. After filtration, the crude was purified by column chromatograph (in ca. 40% yield).

**4,4-Dibenzyloxy-3,5-diphenylpyrazole (3c).** Yellow powder (from MeOH), mp: 150–151 °C; IR (neat, cm^−1^): 3070, 3066, 2948, 2881, 1585, 1557, 1498, 1447, 1391, 1382, 1111, 970, 916, 856, 694; ^1^H NMR (500 MHz, CDCl_3_) δ 4.21 (s, 4H), 7.04–7.06 (m, 4H), 7.19–7.20 (m, 6H), 7.50–7.57 (m, 6H), 8.38–8.39 (m, 4 H); ^13^C NMR (125 MHz, CDCl_3_) δ 66.73 (t, CH_2_), 115.96 (s, C), 127.74 (d, CH, benzyloxy), 127.95 (d, CH, benzyloxy), 128.13 (d, CH, phenyl), 128.18 (d, CH, benzyloxy), 128.26 (d, CH, phenyl), 129.03 (d, CH, phenyl), 132.47 (s, C, phenyl), 135.45 (s, C, benzyloxy), 167.09 (s, C); HRMS–EI calcd for C_29_H_24_N_2_O_2_, 432.1838; found, 432.1842; *R*_f_ = 0.40 (EtOAc/hexane = 30/70).

**4,4-Bis(3,5-dimethoxybenzyloxy)-3,5-diphenylpyrazole (3d).** Yellow powder (from MeOH), mp: 140–141 °C; IR (neat, cm^−1^): 3024, 2951, 2845, 1603, 1475, 1456, 1388, 1160, 1118, 1102, 851; ^1^H NMR (500 MHz, C_6_D_6_) δ 3.19 (s, 12H), 4.21 (s, 4H), 6.28 (d, *J* = 2.36 Hz, 4H), 6.42 (t, *J* = 2.36 Hz, 2H), 7.07 (d, *J* = 8.15 Hz, 6H, phenyl), 8.68 (m, 4H, phenyl); ^13^C NMR (125 MHz, C_6_D_6_) δ 55.76 (q, OCH_3_), 67.11 (t, CH_2_), 100.99 (d, CH, aryl), 106.15 (d, CH, aryl), 116.69 (s, C), 128.35 (d, CH, phenyl), 128.77 (s, C), 129.24 (d, CH, phenyl), 132.30 (d, CH, phenyl), 138.40 (s, C), 161.30 (s, C), 167.29 (s, C); ESIMS (*m*/*z*): [M + Na]^+^ calcd for C_33_H_32_O_6_N_2_Na, 575.21526; found, 575.21387; *R*_f_ = 0.27 (EtOAc/hexane = 30/70).

**4,4-Dibenzyloxy-3,5-bis(3,5-dimethoxyphenyl)pyrazole (3e).** Yellow powder (from MeOH), mp: 183–184 °C; IR (neat, cm^−1^): 3025, 2952, 2847, 1605, 1551, 1456, 1426, 1371, 1160, 1117, 849, 670; ^1^H NMR (500 MHz, C_6_D_6_) δ 3.33 (s, 12H), 4.31 (s, 2H), 6.78 (t, *J* = 2.28 Hz, 2H), 6.93–7.01 (m, 10H), 8.02 (t, *J* = 2.28 Hz, 4H); ^13^C NMR (125 MHz, C_6_D_6_) δ 55.06 (q, OCH_3_), 67.02 (t, CH_2_), 105.54 (d, CH, aryl), 106.52 (d, CH, aryl), 116.22 (s, C), 128.59 (d, CH, phenyl), 128.45 (d, CH, phenyl), 128.19 (d, CH, phenyl), 130.47 (s, C, aryl), 136.24 (s, C, phenyl), 161.81 (s, COCH_3_), 167.53 (s, C); ESIMS (*m*/*z*): [M + H]^+^ calcd for C_33_H_33_N_6_O_2_, 553.23331; found, 553.23267; *R*_f_ = 0.13 (EtOAc/hexane = 20/80).

**4,4-Bis(3,5-dimethoxybenzyloxy)-3,5-bis(3,5-dimethoxyphenyl)pyrazole (3f).** IR (neat, cm^−1^): 3010, 2942, 1605, 1552, 1441, 1371, 1140, 1120, 849; ^1^H NMR (500 MHz, CDCl_3_) δ 3.24 (s, 12H), 3.34 (s, 12H), 4.36 (s, 6H), 6.38 (d, *J* = 2.36 Hz, 4H), 6.43 (t, *J* = 2.36 Hz, 2H), 6.70 (t, *J* = 2.28 Hz, 2H), 8.04 (d, *J* = 2.28 Hz, 4H); ^13^C NMR (125 MHz, C_6_D_6_) δ 54.74 (q, OCH_3_), 55.03 (q, OCH_3_), 67.19 (t, CH_2_), 101.16 (d, CH), 105.71 (d, CH), 106.08 (d, CH), 106.28 (d, CH), 116.36 (s, C), 130.46 (s, C), 138.47 (s, C), 161.33 (s, COCH_3_), 161.77 (s, COCH_3_), 167.58 (s, C); ESIMS (*m*/*z*): [M + Na]^+^ calcd for C_37_H_40_O_10_N_2_Na, 695.25752; found, 695.25775; *R*_f_ = 0.17 (EtOAc/hexane = 30/70).

**4,4-Dimethoxy-3,5-bis(3,5-dimethoxyphenyl)pyrazole (3g).** To a solution of 1,3-bis(3,5-dimethoxyphenyl)-2,2-dimethoxypropane-1,3-dione (2.8 g, 6.92 mmol) in chloroform (10 mL) was added dropwise NH_2_NH_2_·H_2_O (0.40 mL, 8.30 mmol). The mixture was heated under reflux and kept under stirring for 6 h. The reaction was quenched with HCl. A solution of 10% NaHCO_3_ was added to the mixture. After extraction with chloroform, the organic phase was washed with brine, dried with Na_2_SO_4_, concentrated and then purified by column chromatography to give **3g** in 89.6% yield. mp: 179–180 °C; ^1^H NMR (500 MHz, CDCl_3_) δ 3.04 (s, 6H), 3.83 (s, 12H), 6.62 (t, *J* = 2.28 Hz, 2H), 7.40 (d, *J* = 2.28 Hz, 4H); ^13^C NMR (125 MHz, CDCl_3_) δ 51.93 (q, CH_3_), 55.52 (q, OCH_3_), 105.21 (d, CH,), 105.27 (d, CH), 116.91 (s, C), 129.23 (s, C), 161.00 (s, COCH_3_), 166.84 (s, C); IR (neat, cm^−1^): 3012, 2951, 1597, 1548, 1427, 1375, 1158, 1125, 1062, 980, 844; ESIMS (*m*/*z*): calcd for C_21_H_25_N_6_O_2_, 401.17071; found, 401.17041; *R*_f_ = 0.10 (EtOAc/hexane = 20/80).

#### *endo*-2,3-Diazo-10,10-diaryloxy-1,4-diaryltricyclo[5.2.1.0^5,9^]dec-2-ene (**AZc–g**)

**General procedure.** To a solution of cyclopentadiene (1 mL) and pyrazole (2 mmol) in CH_2_Cl_2_ (2 mL) was added dropwise trifluoroacetic acid (1 mmol) in an ice bath under nitrogen. The reaction was traced by TLC analysis. After stirring for about 15 min, the reaction was quenched with 10% aq NaHCO_3_ until the pH of the solution reached 8. After washing with water and brine, the organic phase was dried with MgSO_4_, then filtered and concentrated. The [4 + 2] cycloadduct was dissolved in benzene (2 mL), and 5 mg of PtO_2_ was added as a catalyst. The mixture was stirred under a hydrogen atmosphere for 24 h at room temperature. After stirring, the catalyst was removed by filtration over Celite, and the solvent was evaporated under reduced pressure. The product was purified by column chromatograph to give the product as colorless liquid (ca. 60%). The endo configuration was determined by NOE measurements.

***endo*-2,3-Diazo-10,10-dibenzyloxy-1,4-diphenyltricyclo[5.2.1.0^5,9^]dec-2-ene (AZc).** IR (neat, cm^−1^): 3037, 2968, 2886, 1739, 1607, 1498, 1456, 1387, 1139, 1085, 1029, 702; UV (MTHF) λ_max_ 365 (ε 106.7); ^1^H NMR (500 MHz, C_6_D_6_) δ 1.20–1.75 (m, 6H), 3.69 (t, *J* = 5.13 Hz, 2H), 4.15 (s, 2H), 4.29 (s, 2H), 6.90–8.19 (m, 20H, overlapping with C_6_H_6_); ^13^C NMR (125 MHz, C_6_D_6_) δ 25.96 (t, CH_2_, cyclopentane), 28.16 (t, CH_2_, cyclopentane), 49.27 (d, CH, cyclopentane), 66.16 (t, COCH_2_), 66.34 (t, OCH_2_), 94.83 (s, C), 119.57 (s, C), 126.66 (d, CH), 126.87 (d, CH), 127.33 (d, CH), 128.61 (d, CH), 128.71 (d, CH), 129.01 (d, CH), 137.22 (s, C), 138.13 (s, C), 138.19 (s, C); HRMS–EI (*m*/*z*): calcd for C_34_H_32_O_2_N_2_, 500.6301; found, 500.2462. *R*_f_ = 0.57 (EtOAc/hexane = 20/80).

***endo*-2,3-Diazo-10,10-bis(3,5-dimethoxybenzyloxy)-1,4-diphenyltricyclo[5.2.1.0^5,9^]dec-2-ene (AZd).** IR (neat, cm^−1^): 3022, 2966, 2844, 1751, 1603, 1473, 1326, 1162, 1072, 930, 844, 703; UV (MTHF) λ_max_ 364 (ε 175.1); ^1^H NMR (500 MHz, CDCl_3_) δ 1.25–1.66 (m, 6H), 3.68 (s, 6H), 3.70 (t, *J* = 5.33 Hz, 2H), 3.75 (s, 6H), 3.89 (s, 2H), 4.12 (s, 2H), 6.07 (d, *J* = 2.36 Hz, 2H), 6.24 (t, *J* = 2.36 Hz, 1H), 6.30 (d, *J* = 2.36 Hz, 2H), 6.36 (t, *J* = 2.36 Hz, 1H), 7.40–8.03 (m, 10H); ^13^C NMR (125 MHz, C_6_D_6_) δ 26.21 (t, CH_2_, cyclopentane), 28.34 (t, CH_2_, cyclopentane), 49.42 (d, CH, cyclopentane), 55.69 (q, OCH_3_), 55.74 (q, OCH_3_), 66.17 (t, COCH_2_), 95.15 (s, C), 99.80 (d, CH), 100.00 (d, CH), 104.26 (d, CH), 105.04 (d, CH), 119.54 (s, C), 128.55 (d, CH), 128.92 (d, CH), 128.96 (d, CH), 136.67 (s, C), 140.41 (s, C), 140.67 (s, C), 161.00 (s, C) , 161.28 (s, C); ESIMS (*m*/*z*): [M + H]^+^ calcd for C_38_H_41_O_6_N_2_, 621.29591; found, 621.29449; *R*_f_ = 0.27 (EtOAc/hexane = 20/80).

***endo*-2,3-diazo-1,4-bis(3’,5’-dimethoxybenzyloxy)-10,10-dibenzyloxytricyclo[5.2.1.0^5,9^]dec-2-ene (AZe).** IR (neat, cm^−1^): 3014, 2972, 1600, 1461, 1450, 1357, 1157, 1069, 973, 856; UV (MTHF) λ_max_ 365 (ε 184.2); ^1^H NMR (500 MHz, C_6_D_6_) δ 1.20–1.83 (m, 6H), 3.41 (s, 12H), 3.57 (t, *J* = 4.47 Hz, 2H), 4.31 (s, 2H), 4.46 (s, 2H), 6.68 (t, *J* = 2.28 Hz, 2H), 6.95 (d, *J* = 2.28 Hz, 4H), 7.02–7.57 (m, 10H, overlapping with C_6_D_6_); ^13^C NMR (125 MHz, C_6_D_6_) δ 26.13 (t, CH_2_, cyclopentane), 28.19 (t, CH_2_, cyclopentane), 49.50 (d, CH, cyclopentane), 54.91 (q, OCH_3_), 66.04 (t, COCH_2_), 66.32 (t, COCH_2_), 94.96 (s, C), 101.03 (d, CH), 107.11 (d, CH), 119.74 (s, C), 127.00 (d, CH), 127.16 (d, CH), 127.38 (d, CH), 127.72 (d, CH), 128.54 (d, CH), 128.60 (d, CH), 138.19 (s, C), 138.24 (s, C), 139.58 (s, C), 161.62 (s, C); ESIMS (*m*/*z*): [M + Na]^+^ calcd for C_38_H_40_O_6_N_2_Na, 643.27786; found, 643.27802.

***endo*-2,3-Diazo-1,4-bis(3,5-dimethoxybenzyloxy)-10,10-bis(3,5-dimethoxyphenoxy)tricyclo[5.2.1.0^5,9^]dec-2-ene (AZf).** IR (neat, cm^−1^): 3009, 2965, 2842, 1606, 1467, 1430, 1352, 1160, 1070, 1057, 943, 840; UV (MTHF) λ_max_ 365 (ε 249.6); ^1^H NMR (500 MHz, C_6_D_6_) δ 1.20–1.80 (m, 6H), 3.23 (s, 6H), 3.32 (s, 6H), 3.44 (s, 12H), 3.60 (t, *J* = 5.37 Hz, 2H), 4.38 (s, 2H), 4.56 (s, 2H), 6.33 (d, *J* = 2.29 Hz, 2H), 6.40 (t, *J* = 2.29 Hz, 1H), 6.46 (t, *J* = 2.29 Hz, 1H) 6.54 (d, *J* = 2.29 Hz, 2H), 6.64 (t, *J* = 2.28 Hz, 2H), 7.62 (d, *J* = 2.28 Hz, 4H); ^13^C NMR (125 MHz, C_6_D_6_) δ 26.11 (t, CH_2_, cyclopentane), 28.16 (t, CH_2_, cyclopentane), 49.52 (d, CH, cyclopentane), 54.69 (q, OCH_3_), 54.81 (q, OCH_3_), 54.91 (2×q, OCH_3_), 65.93 (t, COCH_2_), 66.32 (t, COCH_2_), 95.11 (s, C), 100.08 (d, CH), 100.32 (d, CH), 100.92 (2×d, CH), 104.62 (s, C), 104.70 (s, C), 107.23 (2×s, C), 119.91 (s, C), 139.46 (2×s, C), 140.66 (s, C), 140.77 (s, C), 161.33 (2×s, C), 161.58 (s, C), 161.62 (s, C); ESIMS (*m*/*z*): [M + Na]^+^ calcd for C_42_H_48_O_10_N_2_Na, 763.32012; found,763.32043.

***endo*-2,3-Diazo-1,4-bis(3,5-dimethoxybenzyloxy)-10,10-dimethoxytricyclo[5.2.1.0^5,9^]dec-2-ene (AZg).** IR (neat, cm^−1^): 2973, 2846, 1602, 1464, 1359, 1158, 1088, 1022, 942, 848; UV (MTHF) λ_max_ 364 (ε 169.9); ^1^H NMR (500 MHz, C_6_D_6_) δ 0.9–1.75 (m, 6H), 2.69 (s, 3H), 2.80 (s, 3H), 3.36 (t, *J* = 5.49 Hz, 2H), 3.42 (s, 12H), 6.64 (t, *J* = 2.28 Hz, 2H), 7.48 (d, *J* = 2.28 Hz, 4H); ^13^C NMR (125 MHz, C_6_D_6_) δ 26.08 (t, CH_2_, cyclopentane), 28.16 (t, CH_2_, cyclopentane), 49.29 (d, CH, cyclopentane), 51.46 (q, OCH_3_), 51.75 (q, OCH_3_), 51.92 (2×q, OCH_3_), 94.65 (s, C), 100.30 (d, CH), 107.33 (d, CH), 119.76 (s, C), 139.86 (s, C), 161.52 (s, C); ESIMS (*m*/*z*): [M + Na]^+^ calcd for C_26_H_32_O_6_N_2_Na, 491.21526; found, 491.21466.

**General procedure for photolysis.** A sample (30.0 mg) of the diazenes **AZ** was dissolved in 1.0 mL of C_6_D_6_. The photolysis was performed with a 500 W Xenon-lamp through a Pyrex filter (*h*ν > 300 nm) at room temperature (ca. 20 °C). The photolysate was directly analyzed by NMR spectroscopy (^1^H: 500 MHz, ^13^C: 125 MHz), which indicated the quantitative formation of the housanes **CP**. The housanes **CPc–g** were isolated by using silica-gel column chromatography. The spectroscopic data are as follows:

**3,3-Dibenzyloxy-2,4-diphenyltricyclo[3.3.0.0^2,4^]octane (CPc). **^1^H NMR (500 MHz, C_6_D_6_) δ 1.41–1.93 (m, 6H), 3.19 (d, *J* = 6.34 Hz, 2H), 4.31 (s, 2H), 4.92 (s, 2H), 6.96–7.45 (m, 20H, overlapping with C_6_D_6_); ^13^C NMR (125 MHz, C_6_D_6_) δ 25.28 (t, CH_2_, cyclopentane), 28.38 (t, CH_2_, cyclopentane), 41.73 (d, CH, cyclopentane), 48.05 (s, C), 67.16 (t, COCH_2_), 69.66 (t, OCH_2_), 98.43 (s, C), 126.57 (d, CH), 127.26 (d, CH), 127.40 (d, CH), 127.92 (d, CH), 128.12 (d, CH), 128.35 (d, CH), 128.46 (d, CH), 128.68 (d, CH), 130.54 (d, CH), 135.25 (s, C, phenyl), 138.59 (s, C, benzyloxy), 138.90 (s, C, benzyloxy); HRMS–EI (*m*/*z*): calcd for C_34_H_32_O_2_, 472.2402; found, 472.2424.

**3,3-Bis(3,5-dimethoxybenzyloxy)-2,4-diphenyltricyclo[3.3.0. 0^2,4^]octane (CPd). **^1^H NMR (500 MHz, CDCl_3_) δ 1.40–1.92 (m, 6H), 3.12 (d, *J* = 6.29 Hz, 2H), 3.22 (s, 6H), 3.37 (s, 6H), 4.35 (s, 2H), 4.99 (s, 2H), 6.31 (d, *J* = 2.36 Hz, 2H), 6.41 (t, *J* = 2.36 Hz, 1H), 6.56 (d, *J* = 2.36 Hz, 1H), 6.81 (d, *J* = 2.36 Hz, 2H), 7.02–7.46 (m, 10H, overlapping with C_6_D_6_); ^13^C NMR (125 MHz, C_6_D_6_) δ 25.22 (t, CH_2_, cyclopentane), 28.34 (t, CH_2_, cyclopentane), 41.68 (d, CH, cyclopentane), 48.18 (s, C), 54.68 (q, OCH_3_), 54.89 (q, OCH_3_), 67.07 (t, COCH_2_), 69.78 (t, COCH_2_), 98.46 (s, C), 100.04 (d, CH), 100.06 (d, CH), 104.91 (d, CH), 105.94 (d, CH), 126.48 (2×d, CH, phenyl), 128.04 (2×d, CH, phenyl), 130.51 (2×d, CH, phenyl), 135.22 (2×s, C, phenyl), 140.90 (s, C), 141.30 (s, C), 161.19 (s, COCH_3_), 161.64 (s, COCH); ESIMS (*m*/*z*): [M + Na]^+^ calcd for C_38_H_41_O_6_Na, 615.27171; found, 615.27130.

**3,3-Bisbenzyloxy-2,4-bis(3,5-dimethoxyphenyl)tricyclo[3.3.0.0^2,4^]octane (CPe). **^1^H NMR (500 MHz, C_6_D_6_) δ 1.40–2.02 (m, 6H), 3.05 (d, *J* = 6.40 Hz, 2H), 3.29 (s, 12H), 4.41 (s, 2H), 4.90 (s, 2H), 6.43 (t, *J* = 2.28 Hz, 2H), 6.80 (d, *J* = 2.28 Hz, 4H), 6.86–7.34 (m, 10H, overlapping with C_6_D_6_); ^13^C NMR (125 MHz, C_6_D_6_) δ 25.44 (t, CH_2_, cyclopentane), 28.43 (t, CH_2_, cyclopentane), 41.82 (d, CH, cyclopentane), 48.47 (s, C), 54.78 (q, OCH_3_), 67.44 (t, COCH_2_), 69.51 (t, COCH_2_), 98.39 (s, C), 99.22 (d, CH), 108.88 (d, CH), 127.19 (d, CH), 127.54 (d, CH), 127.80 (d, CH), 128.06 (d, CH), 128.18 (d, CH), 128.59 (d, CH), 137.30 (s, C), 138.50 (s, C, phenyl), 138.81 (s, C, phenyl), 160.97 (s, C); ESIMS (*m*/*z*): [M + Na]^+^ calcd for C_38_H_40_O_6_Na, 615.27171; found, 615.27167.

**3,3-Bis(3,5-dimethoxybenzyloxy)-2,4-bis(3,5-dimethoxyphenyl)tricyclo[3.3.0.0^2,4^]octane (CPf). **^1^H NMR (500 MHz, C_6_D_6_) δ 1.43–2.08 (m, 6H), 3.15 (d, *J* = 6.17 Hz, 2H), 3.30 (s, 6H), 3.35 (s, 12H), 3.38 (s, 6H), 4.51 (s, 2H), 5.01 (s, 2H), 6.34 (d, *J* = 2.36 Hz, 2H), 6.41 (t, *J* = 2.36 Hz, 1H), 6.47 (t, *J* = 2.28 Hz, 2H), 6.54 (t, *J* = 2.36 Hz, 1H), 6.80 (d, *J* = 2.36 Hz, 2H), 6.87 (d, *J* = 2.28 Hz, 4H); ^13^C NMR (125 MHz, C_6_D_6_) δ 25.46 (t, CH_2_, cyclopentane), 28.44 (t, CH_2_, cyclopentane), 41.80 (d, CH, cyclopentane), 48.64 (s, C), 54.67 (q, OCH_3_), 54.78 (q, OCH_3_), 54.89 (2×q, OCH_3_), 67.29 (t, COCH_2_), 69.76 (t, COCH_2_), 98.41 (s, C), 99.18 (2×d, CH), 100.12 (d, CH), 100.32 (d, CH), 104.93 (d, CH), 105.96 (d, CH), 108.89 (2×d, CH), 137.30 (2×s, C), 140.88 (s, C), 141.26 (s, C), 160.96 (2×s, COCH_3_), 161.187 (s, COCH_3_), 161.59 (s, COCH_3_); ESIMS (*m*/*z*): [M + Na]^+^ calcd for C_42_H_48_O_10_Na, 735.31397; found,735.31415.

**3,3-Dimethoxy-2,4-bis(3’,5’-dimethoxyphenyl)tricyclo[3.3.0.0^2,4^]octane (CPg). **^1^H NMR (500 MHz, C_6_D_6_) δ 1.43–2.03 (m, 6H), 2.94 (s, 3H), 2.96 (d, *J* = 6.47 Hz, 2H), 3.37 (s, 12H), 3.48 (s, 3H), 6.51 (t, *J* = 2.28 Hz, 2H), 6.79 (d, *J* = 2.28 Hz, 4H); ^13^C NMR (125 MHz, C_6_D_6_) δ 25.49 (t, CH_2_, cyclopentane), 28.45 (t, CH_2_, cyclopentane), 41.83 (d, CH, cyclopentane), 48.13 (s, C), 52.37 (q, OCH_3_), 54.01 (q, OCH_3_), 54.85 (2×q, OCH_3_), 98.61 (s, C), 99.05 (d, CH), 108.88 (d, CH), 137.59 (s, COCH_3_), 161.07 (s, C); ESIMS (*m*/*z*): [M + Na]^+^ calcd for C_26_H_32_O_6_Na, 463.20911; found, 463.20844.

## Supporting Information

File 1 NMR spectra of compounds **1**–**5**, **AZc**–**g**, and **CPc**–**g**.
