# Supplementary material for: Substituent effect on the energy barrier for σ-bond formation from π-single-bonded species, singlet 2,2-dialkoxycyclopentane-1,3-diyls
Source: Beilstein J Org Chem. 2013 May 14;9:925–33. doi: 10.3762/bjoc.9.106 (PMC3678856; doi:10.3762/bjoc.9.106)

Supporting Information

for

Substituent effect on the energy barrier for σ-bond formation from π-single-bonded species, singlet 2,2-dialkoxycyclopentane-1,3-diyls

Jianhuai Ye[1], Yoshihisa Fujiwara[2], and Manabu Abe*[1,3]

Address: [1]Department of Chemistry, Graduate School of Science, Hiroshima University, Japan, [2]Department of Mathematical and Life Sciences, Graduate School of Science, Hiroshima University, Japan, and [3]Institute for Molecular Science (IMS), Okazaki, Aichi 444-8787, Japan

Email: Manabu Abe - mabe@hiroshima-u.ac.jp

* Corresponding author

NMR spectra of compounds **1–5**, **AZc–g**, and **CPc–g**

3,5-Dimethoxyphenyl-[1,2,4,5]tetrazine (1f) ^{1}H NMR

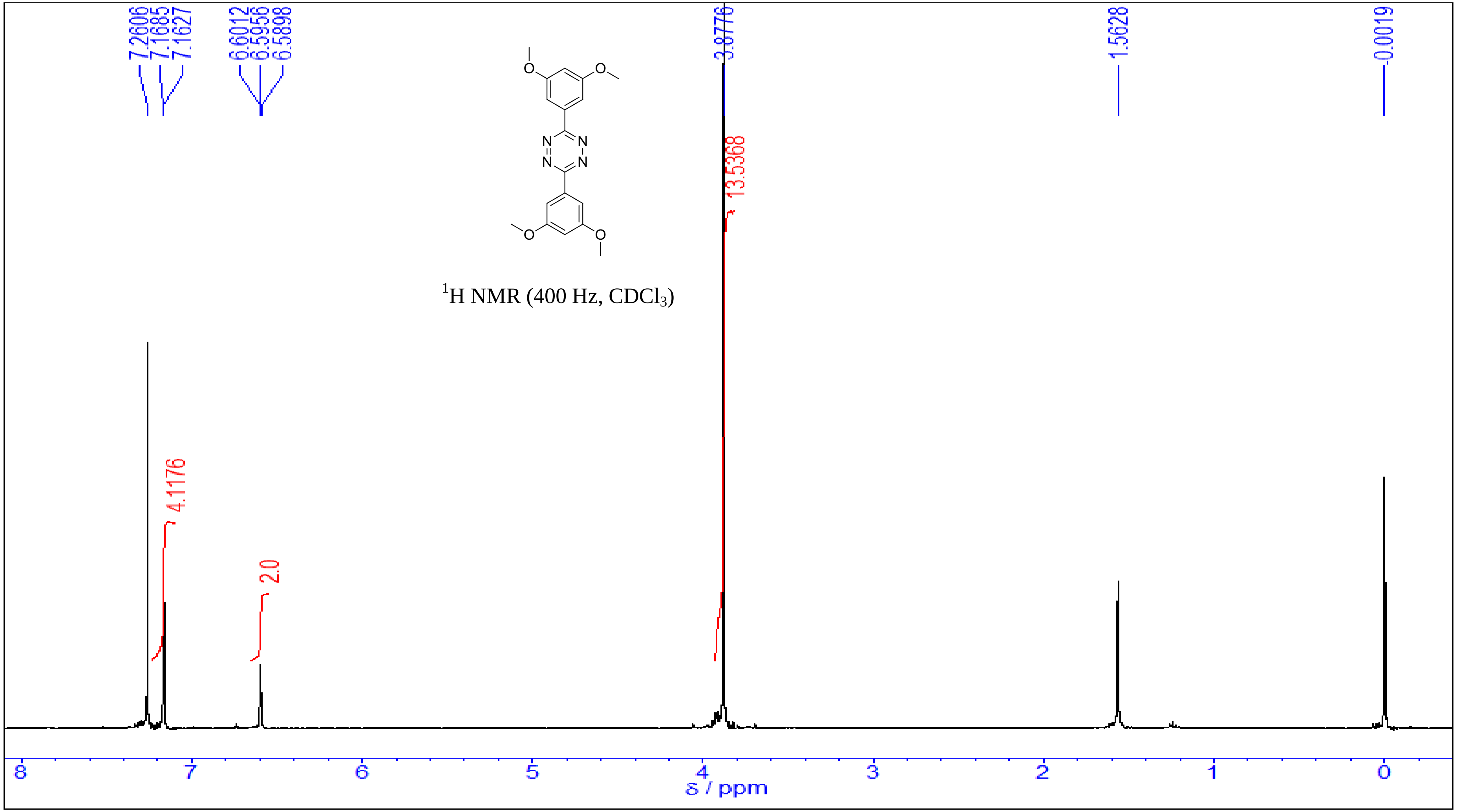

3,5-Dimethoxyphenyl-[1,2,4,5]tetrazine (1f) ^{13}C NMR

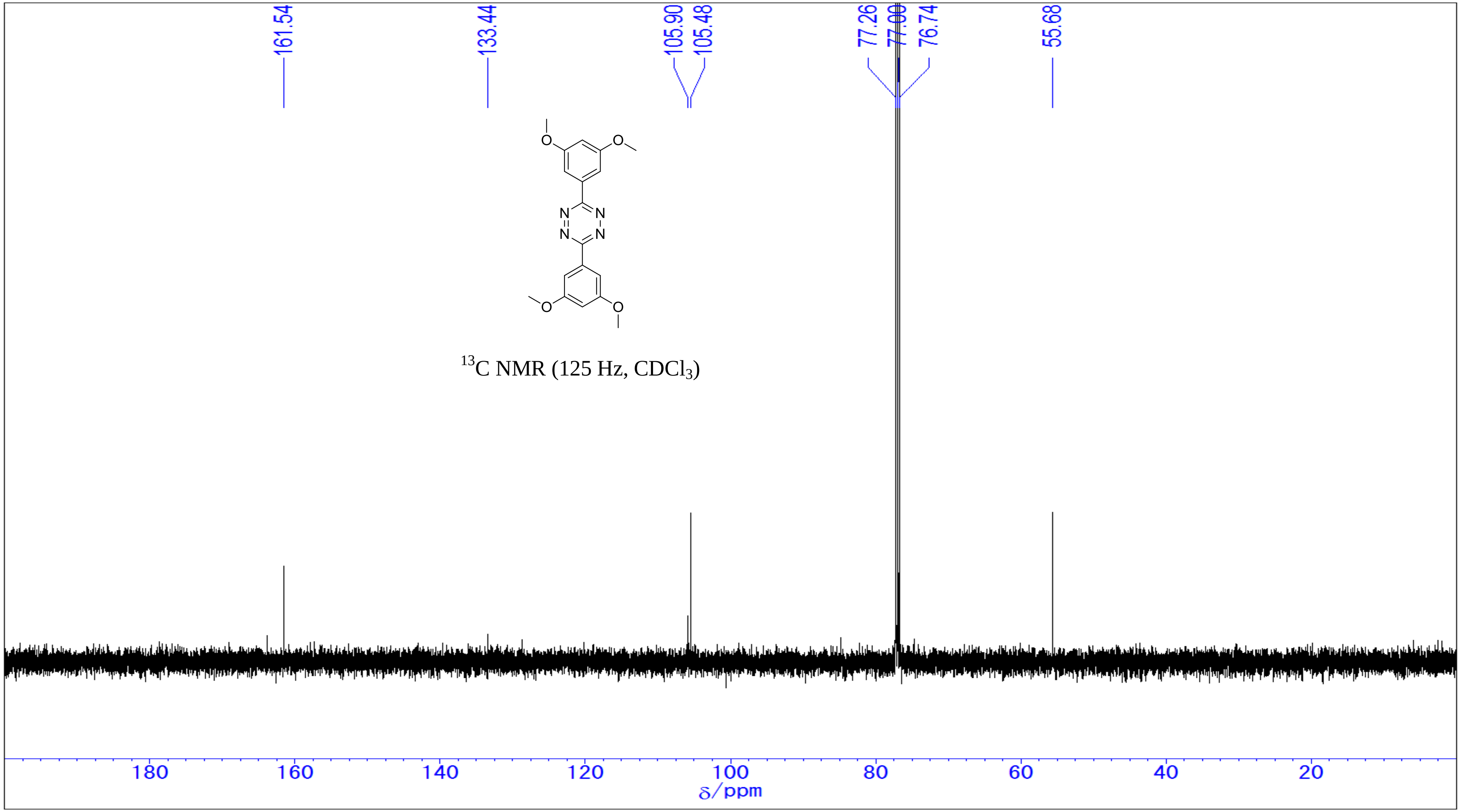

5,5-Dimethyl-2,2-bis(3,5-dimethoxybenzyl)-Δ^3-1,3,4-oxadiazoline (2f) ^{1}H NMR

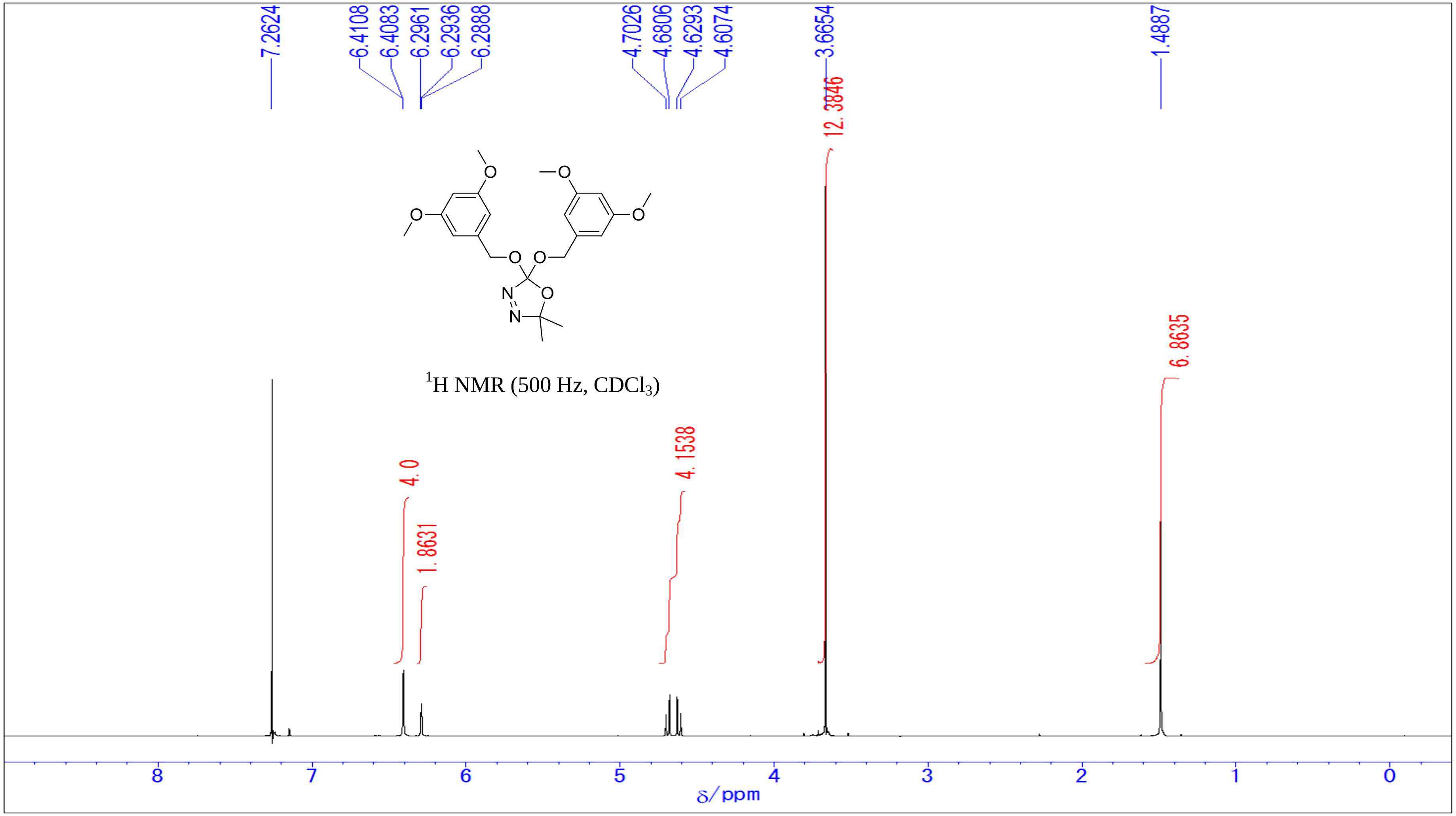

5,5-Dimethyl-2,2-bis(3,5-dimethoxybenzyl)-Δ^3-1,3,4-oxadiazoline (2f) ^{13}C NMR

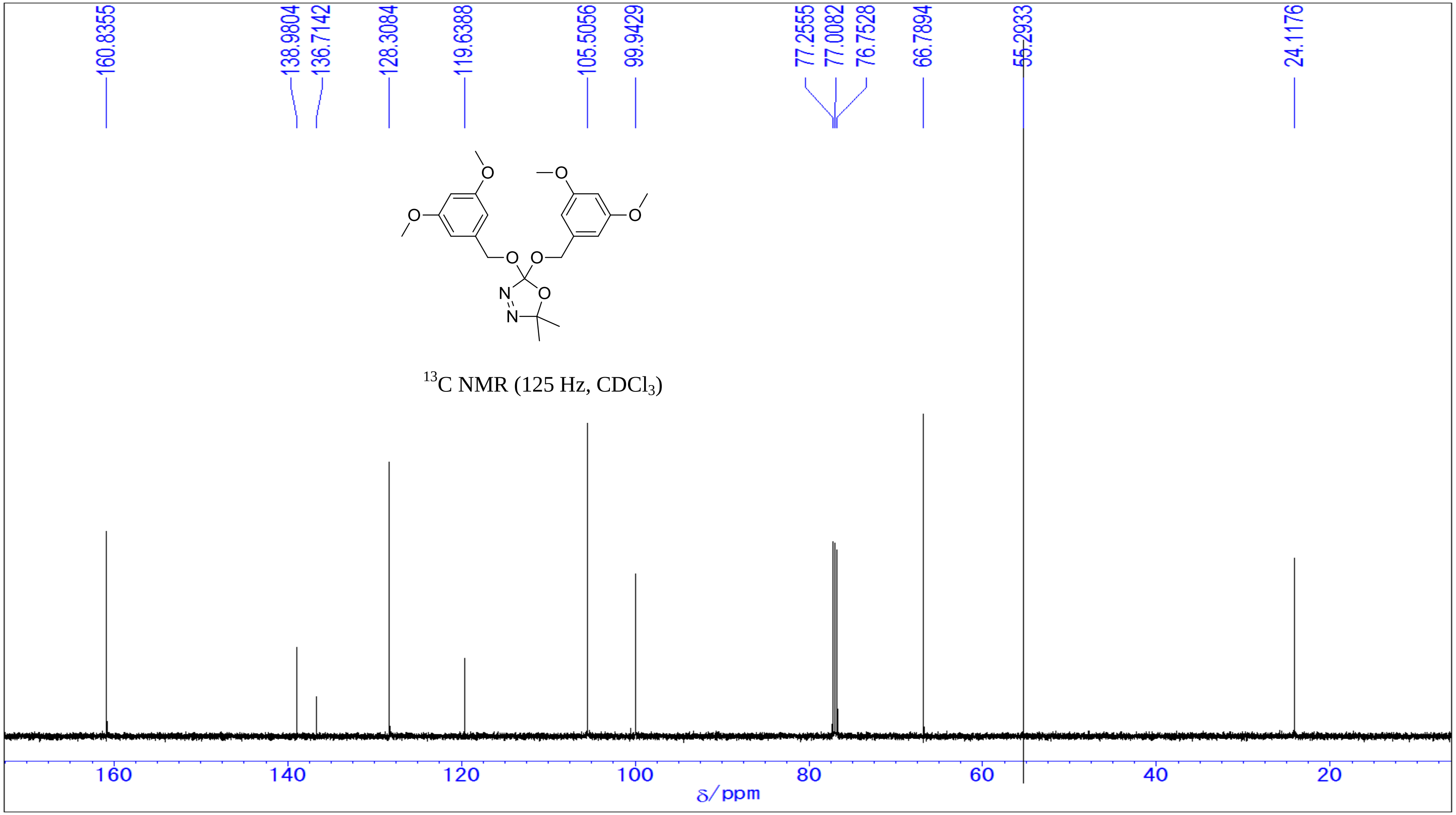

1,3-Bis(3,5-dimethoxyphenyl)propane-1,3-dione (4) ^{1}H NMR

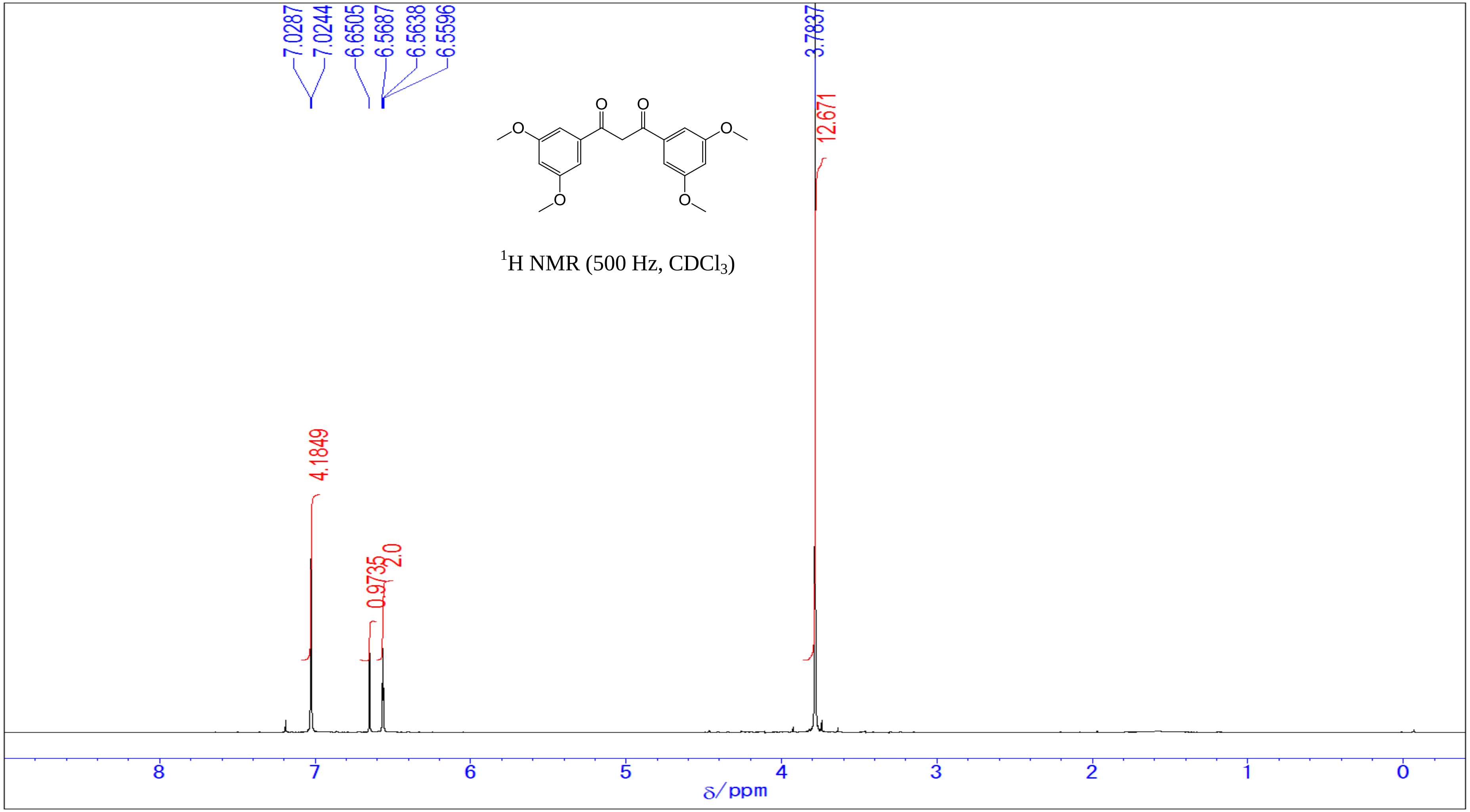

1,3-Bis(3,5-dimethoxyphenyl)propane-1,3-dione (4) ^{1}H NMR

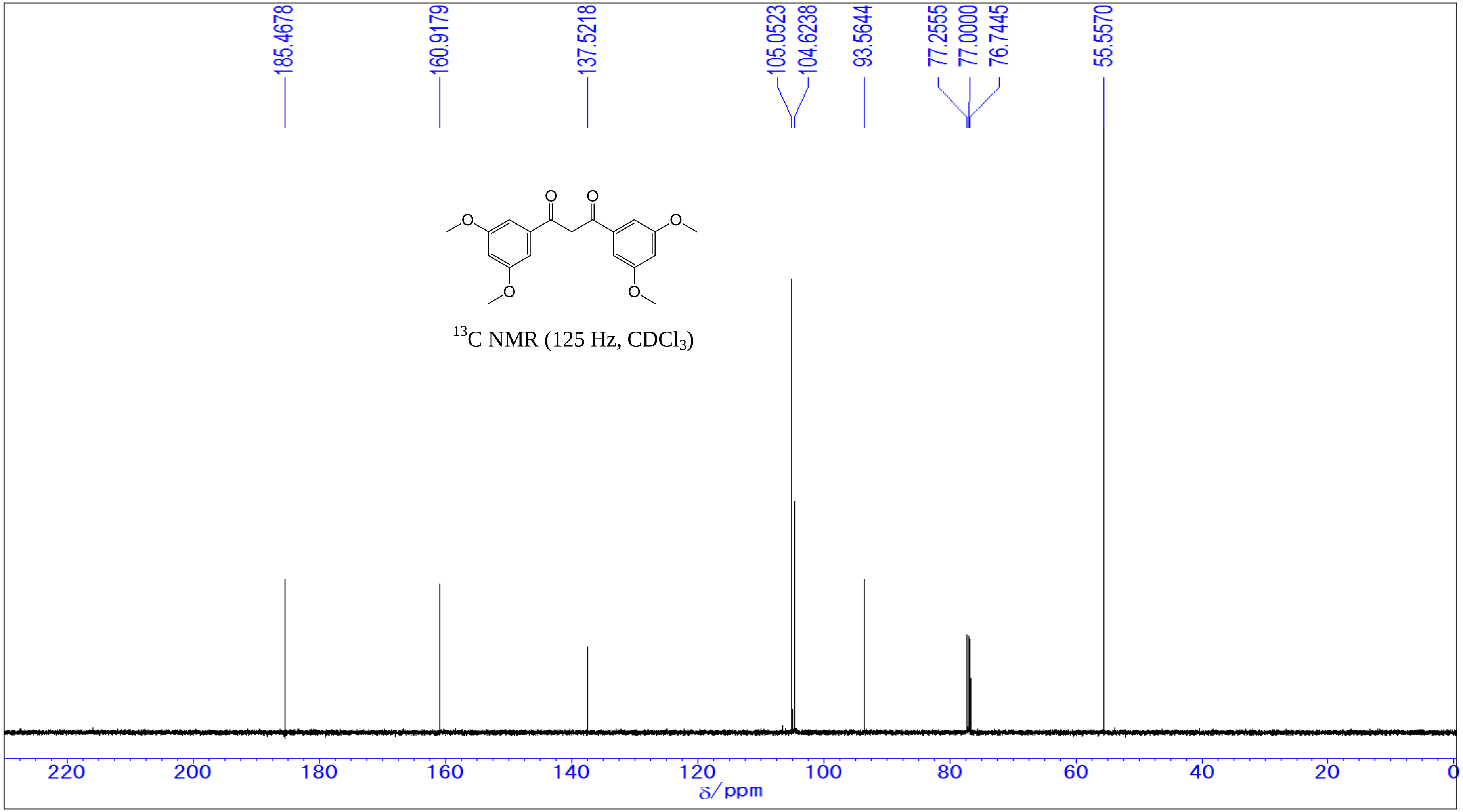

1,3-Bis(3,5-dimethoxyphenyl)-2,2-dimethoxypropane-1,3-dione (5) ^{13}H NMR

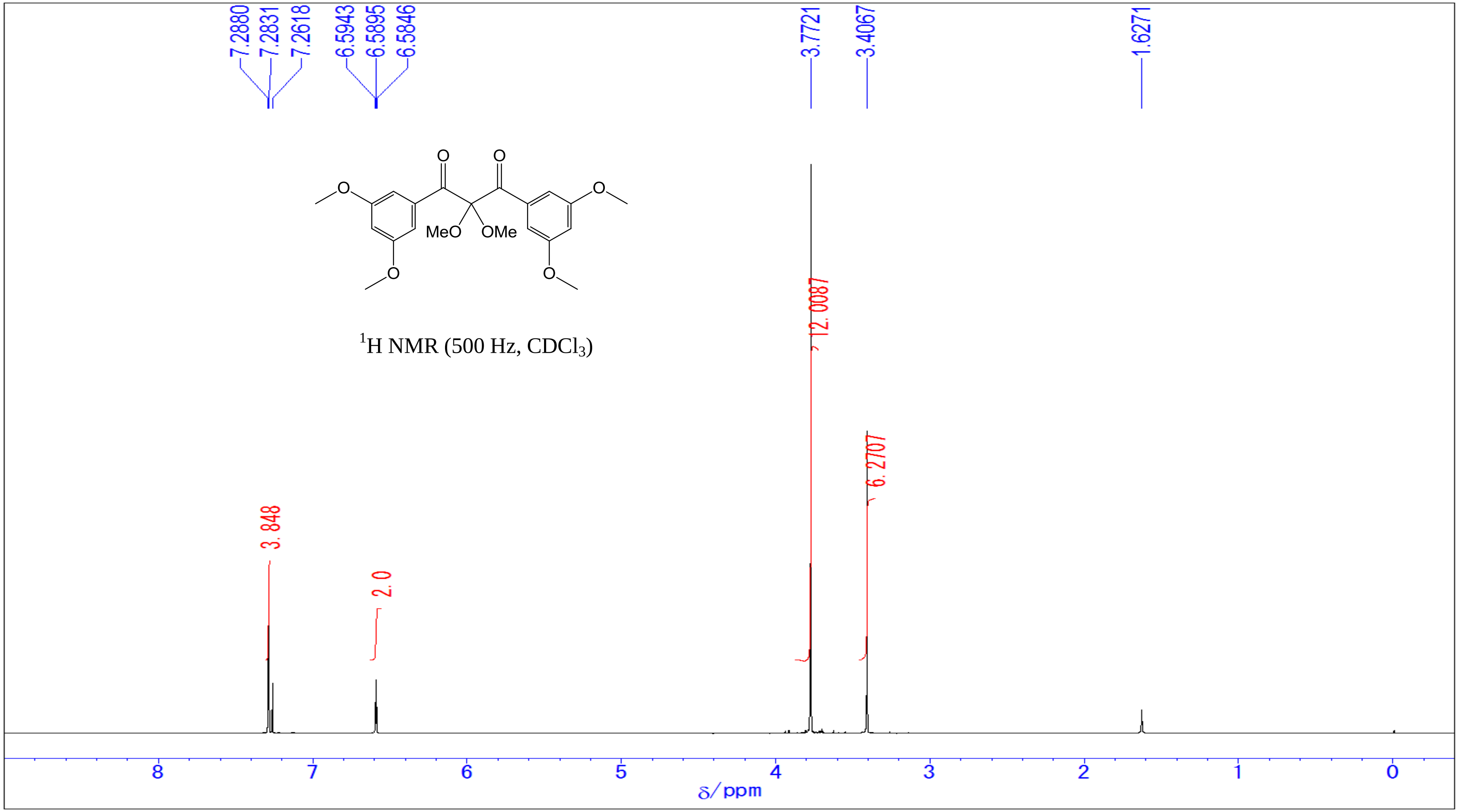

1,3-Bis(3,5-dimethoxyphenyl)-2,2-dimethoxypropane-1,3-dione (5) ^{13}C NMR

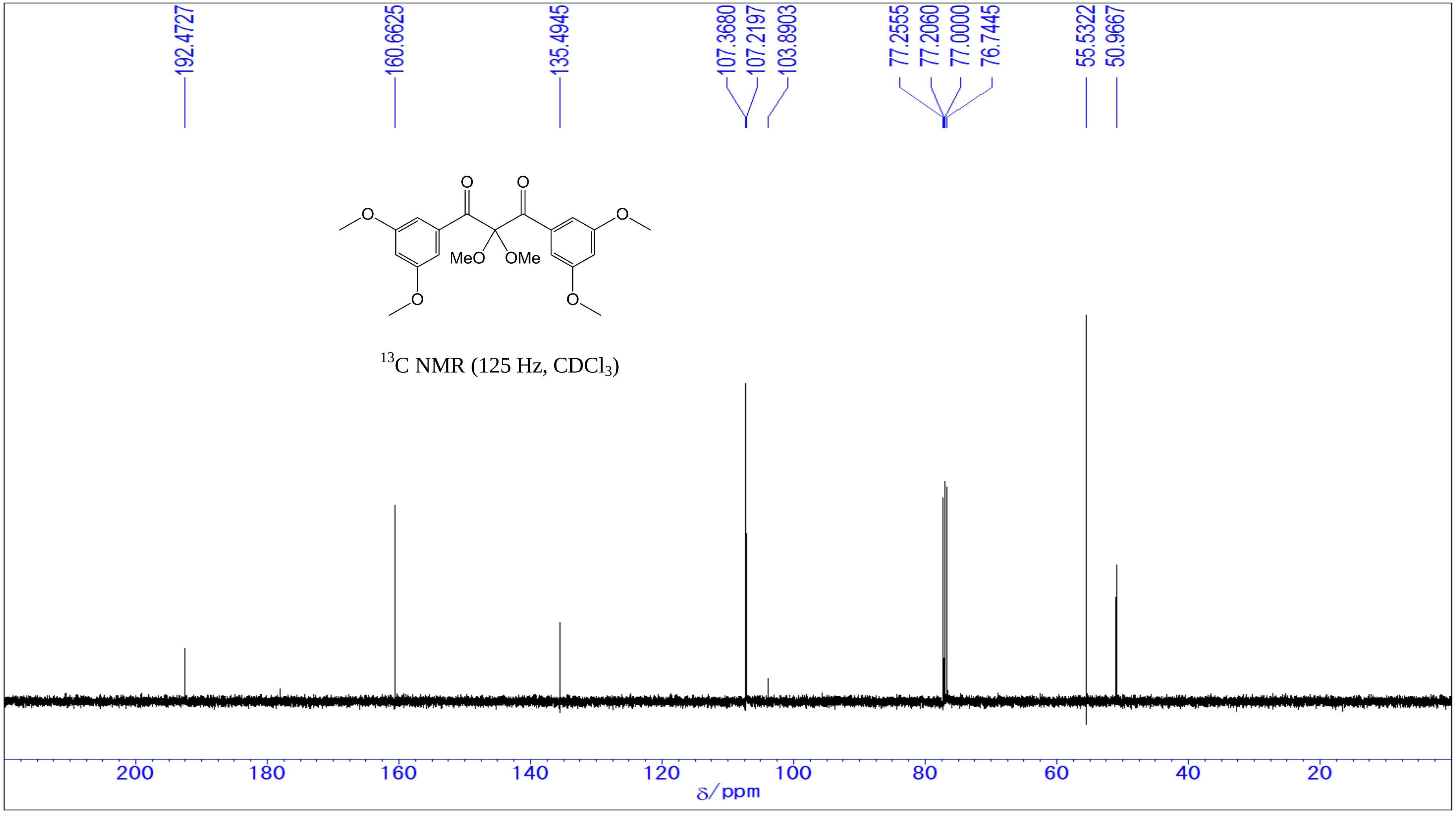

4,4-Dibenzyloxy-3,5-diphenylpyrazole (3c) ^{1}H NMR

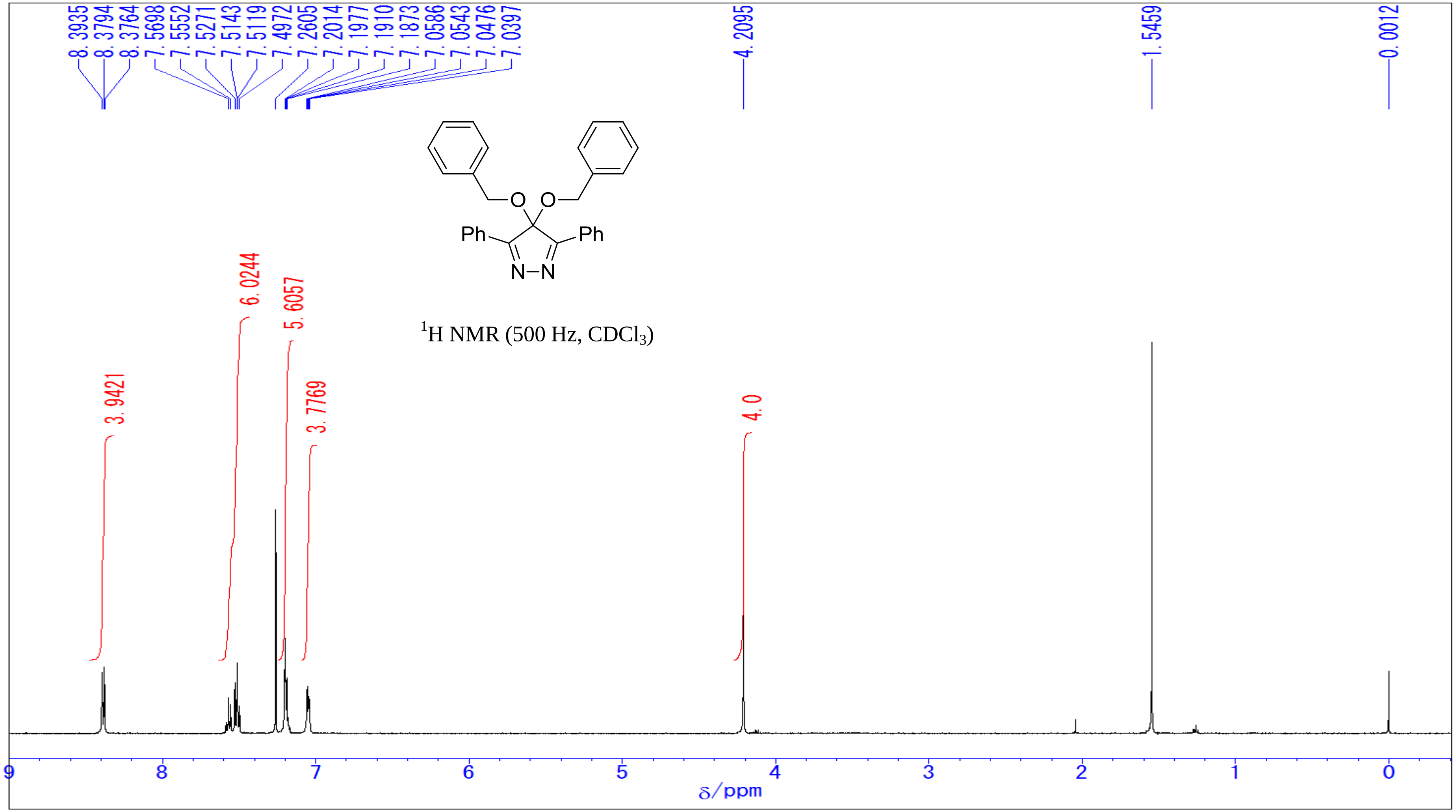

4,4-Dibenzyloxy-3,5-diphenylpyrazole (3c) ^{13}C NMR

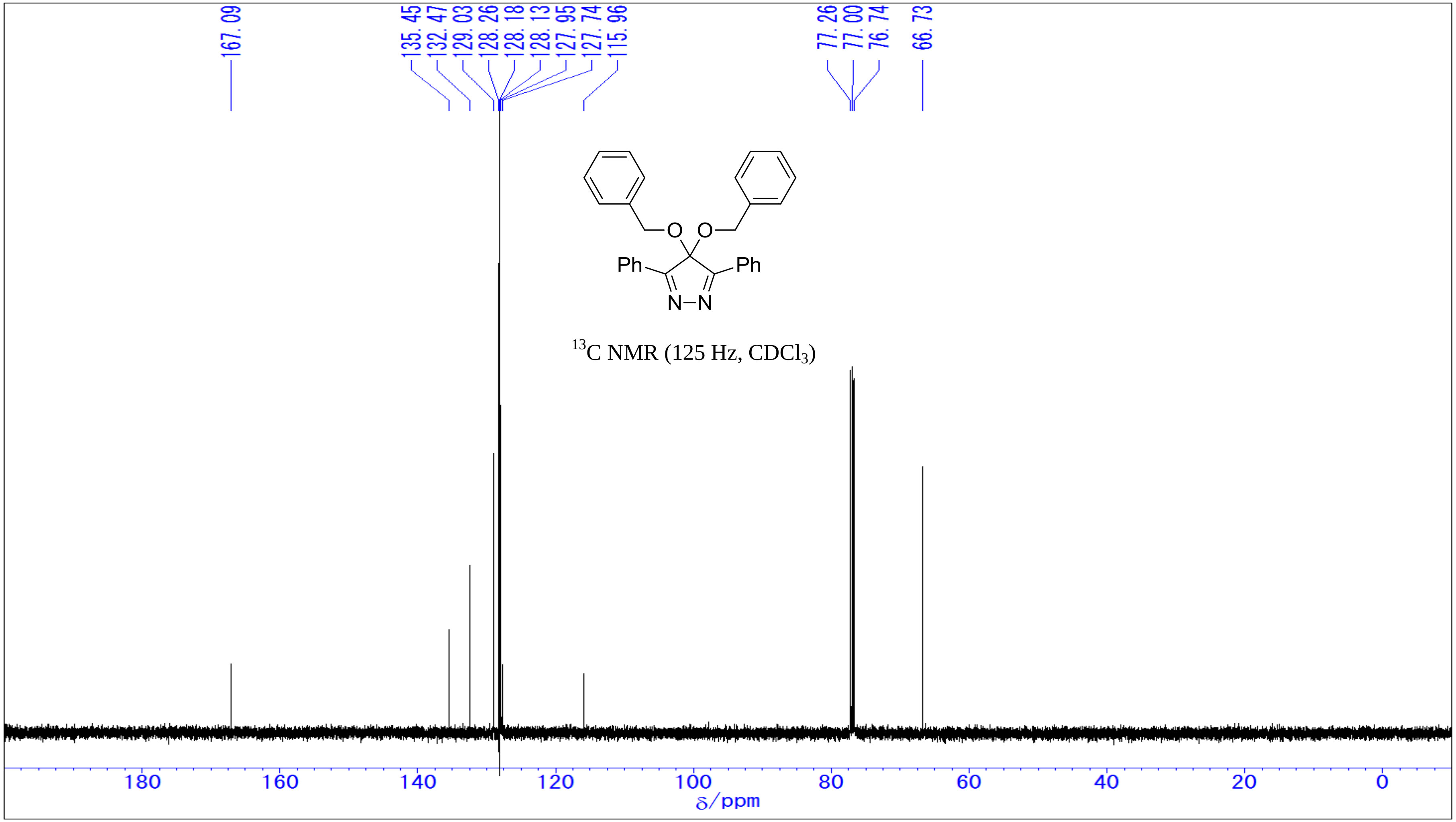

4,4-Bis(3,5-dimethoxybenzyloxy)-3,5-diphenylpyrazole (3d) ^{1}H NMR

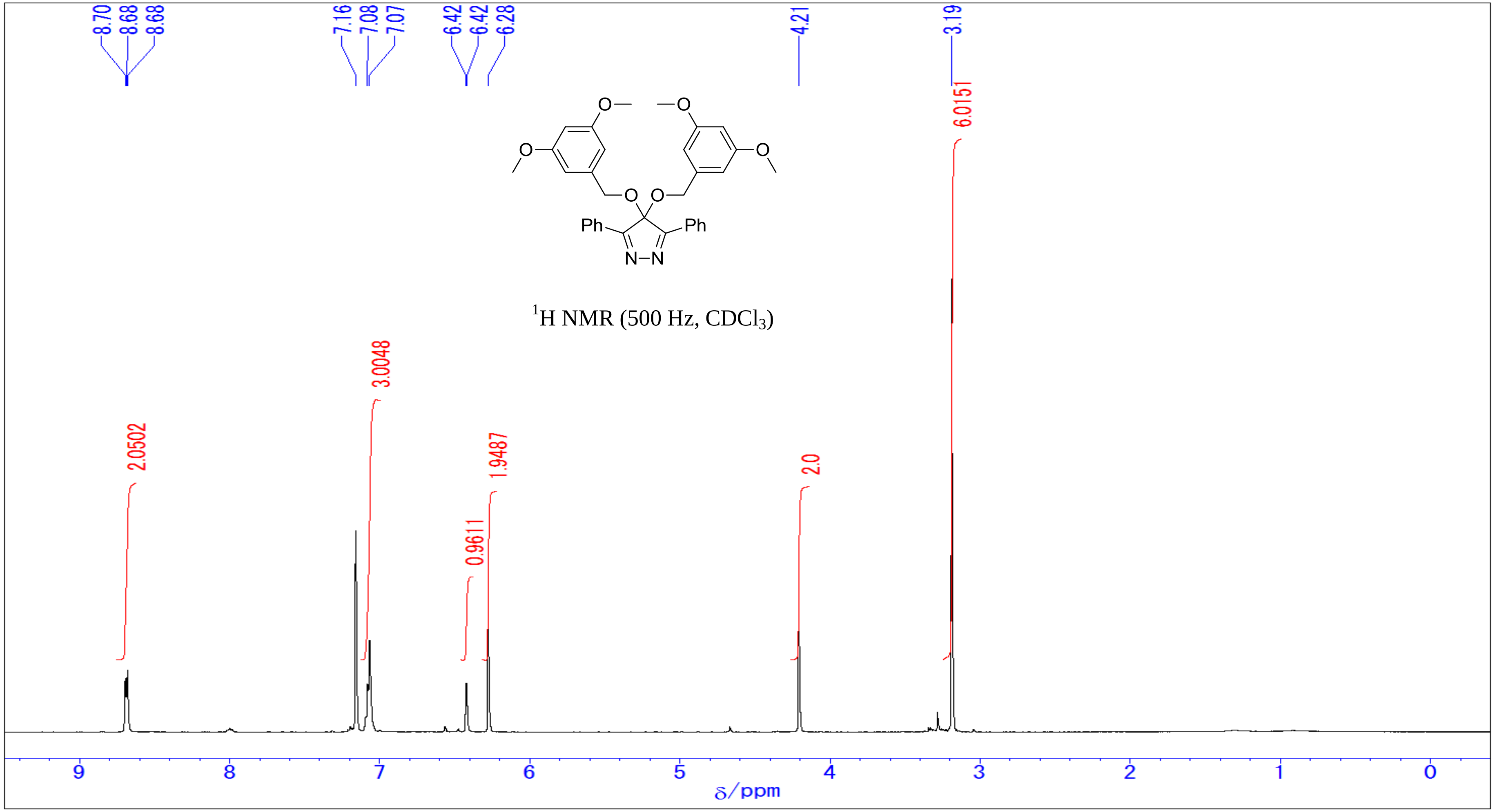

4,4-Bis(3,5-dimethoxybenzyloxy)-3,5-diphenylpyrazole (3d) ^{13}C NMR

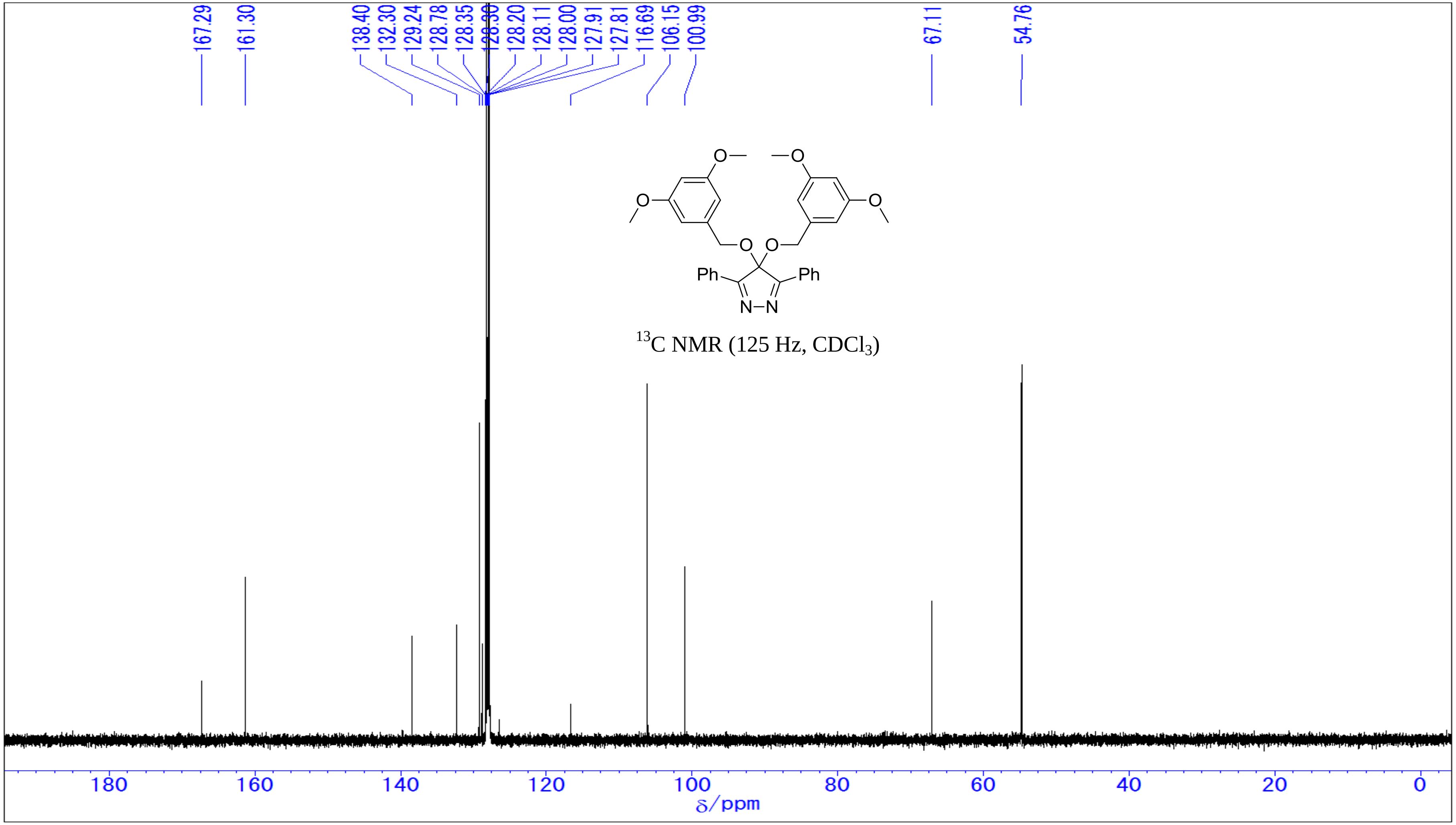

4,4-Dibenzyloxy-3,5-bis(3,5-dimethoxyphenyl)pyrazole (3e) ^{1}H NMR

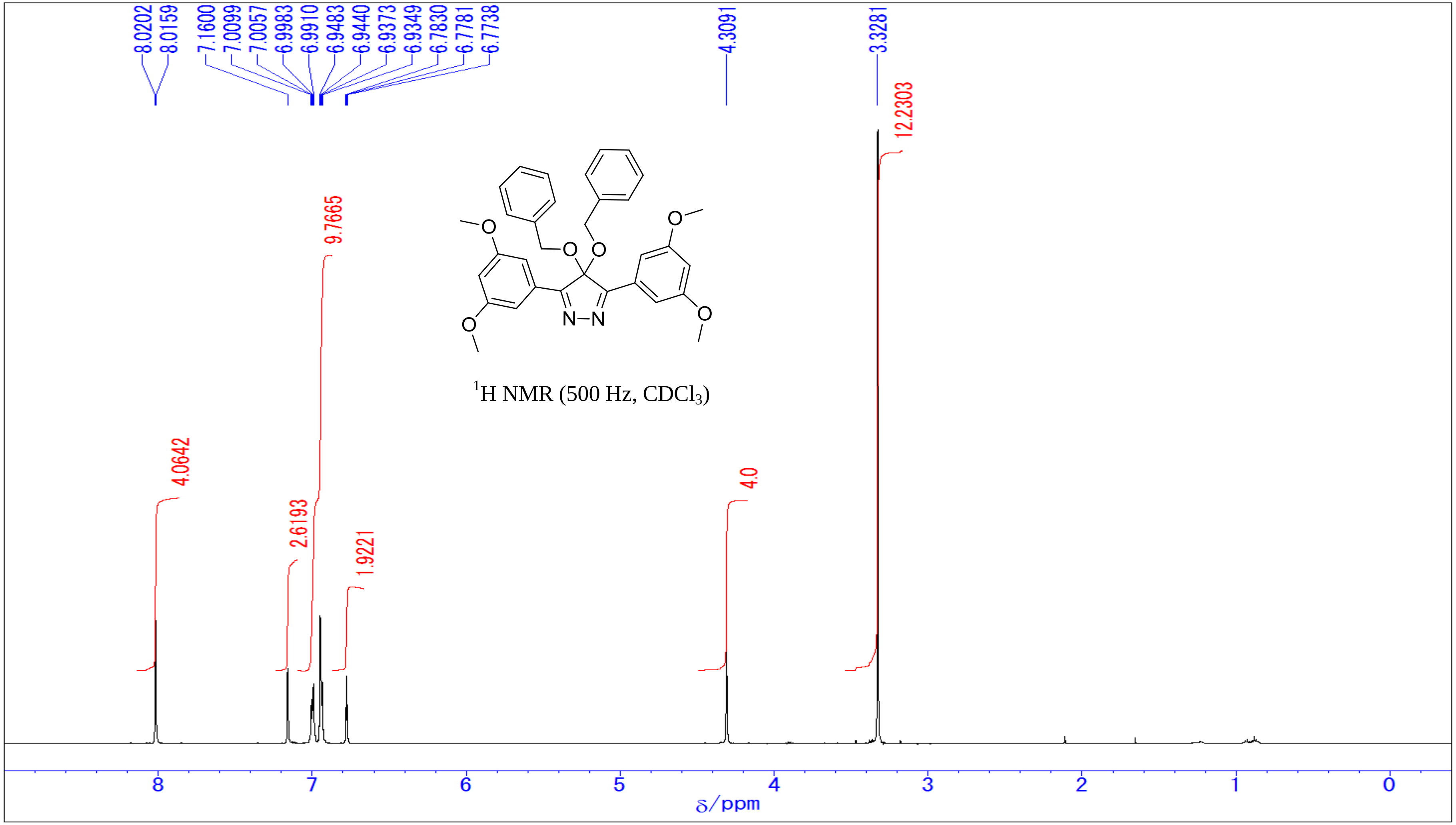

4,4-Dibenzyloxy-3,5-bis(3,5-dimethoxyphenyl)pyrazole (3e) ^{13}C NMR

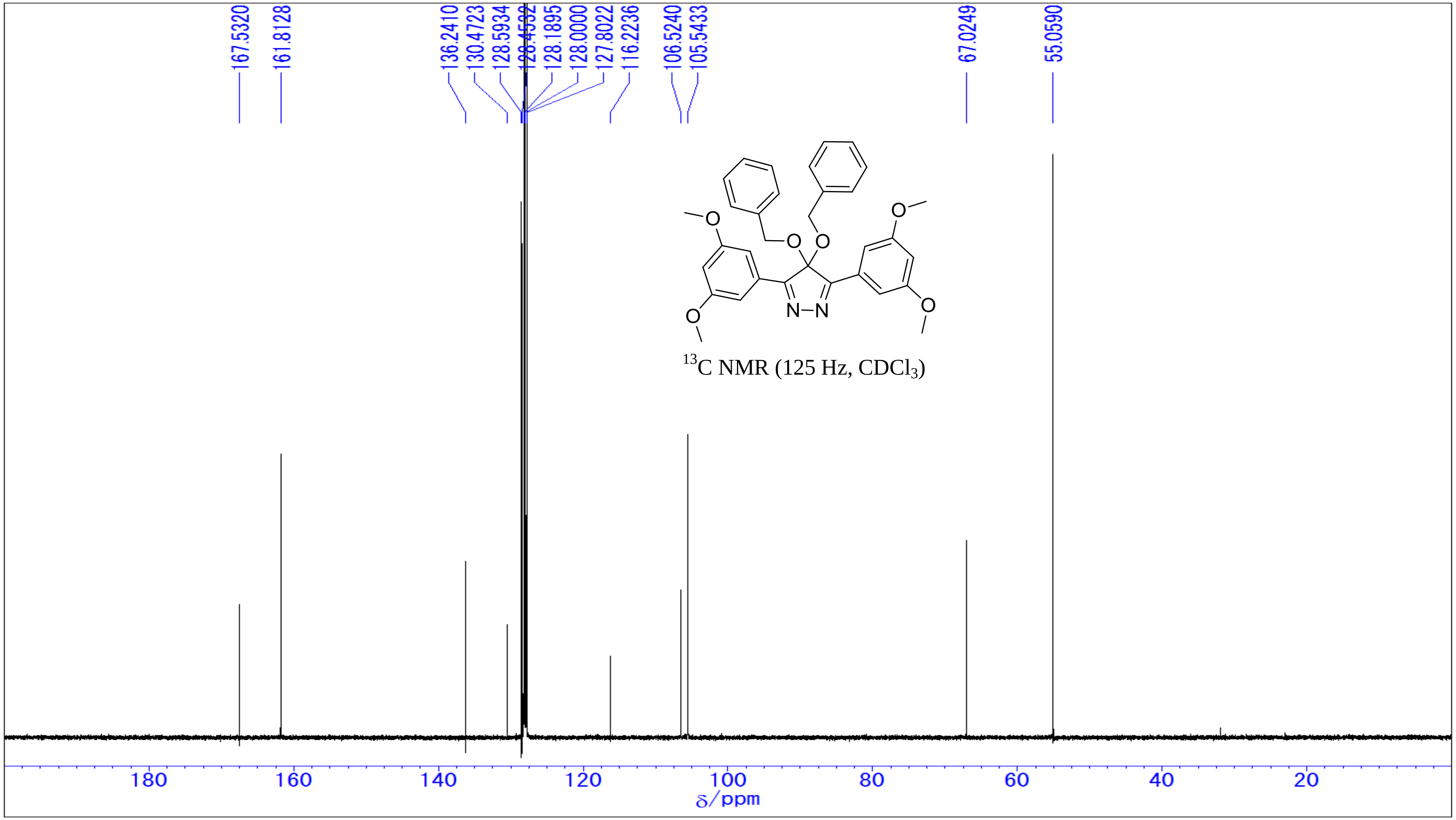

4,4-Bis(3,5-dimethoxybenzyloxy)-3,5-bis(3,5-dimethoxyphenyl)pyrazole (3f) ^{1}H NMR

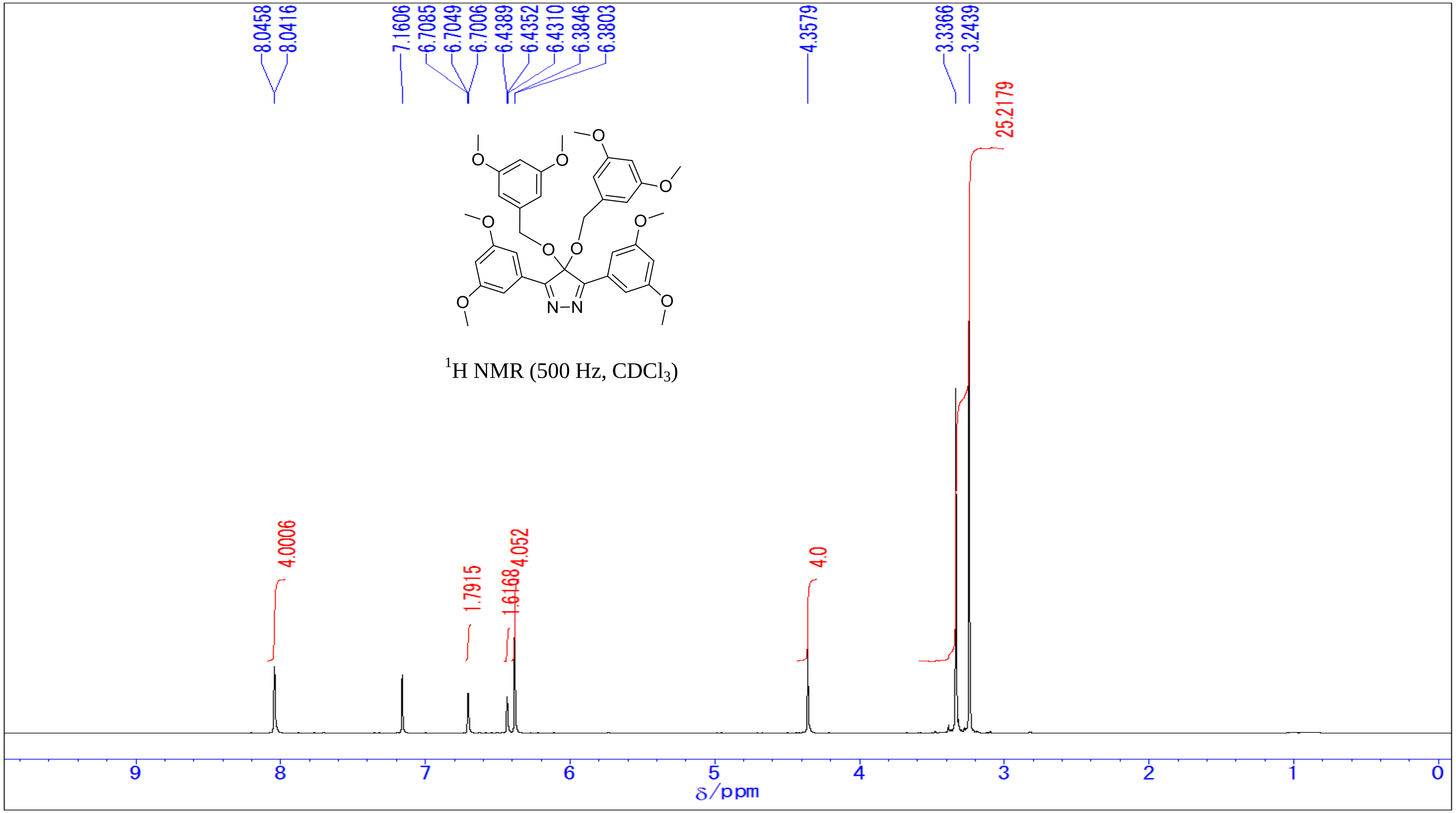

4,4-Bis(3,5-dimethoxybenzyloxy)-3,5-bis(3,5-dimethoxyphenyl)pyrazole (3f) ^{13}C NMR

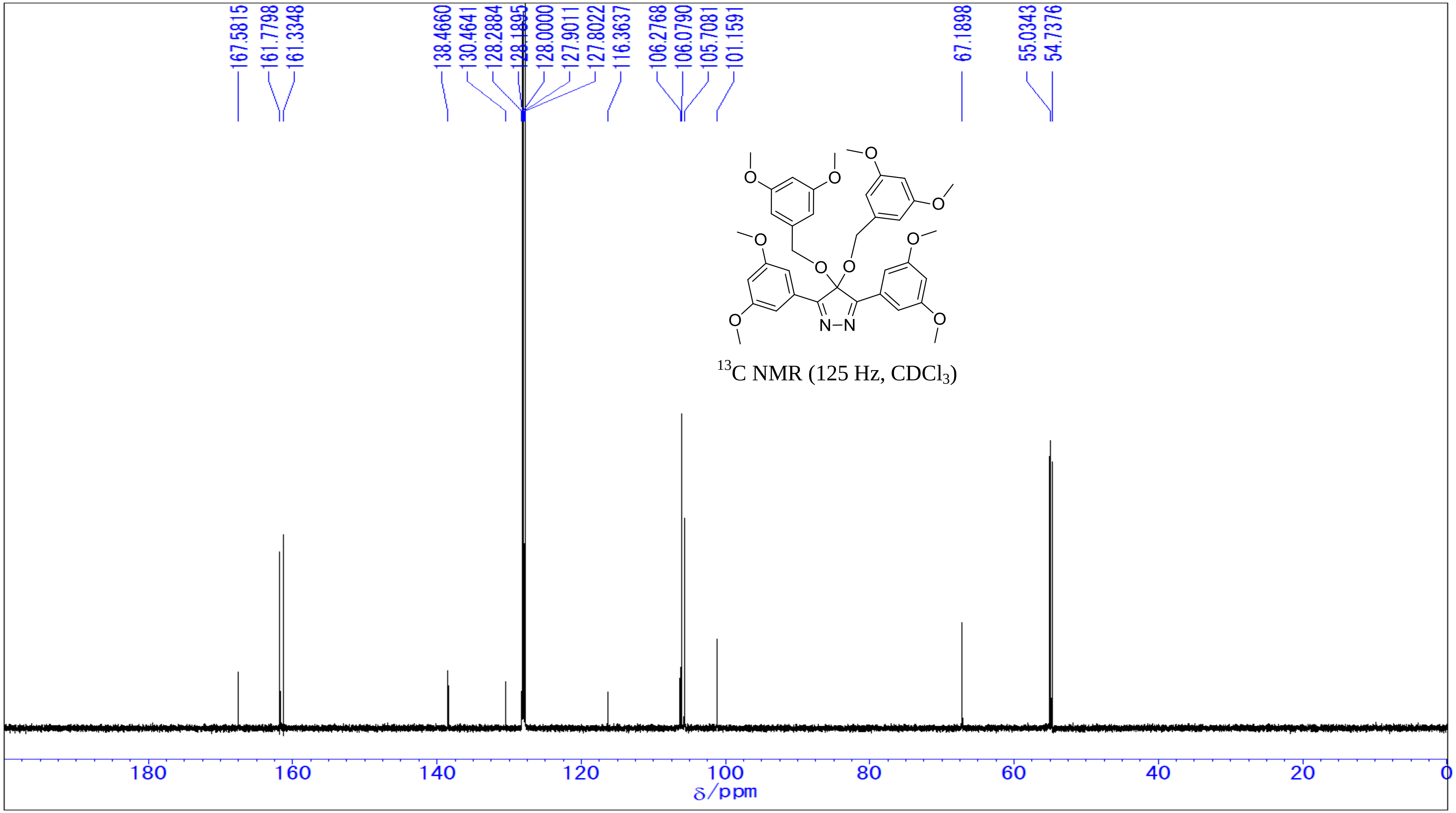

4,4-Dimethoxy-3,5-bis(3,5-dimethoxyphenyl)pyrazole (3g) ^{1}H NMR

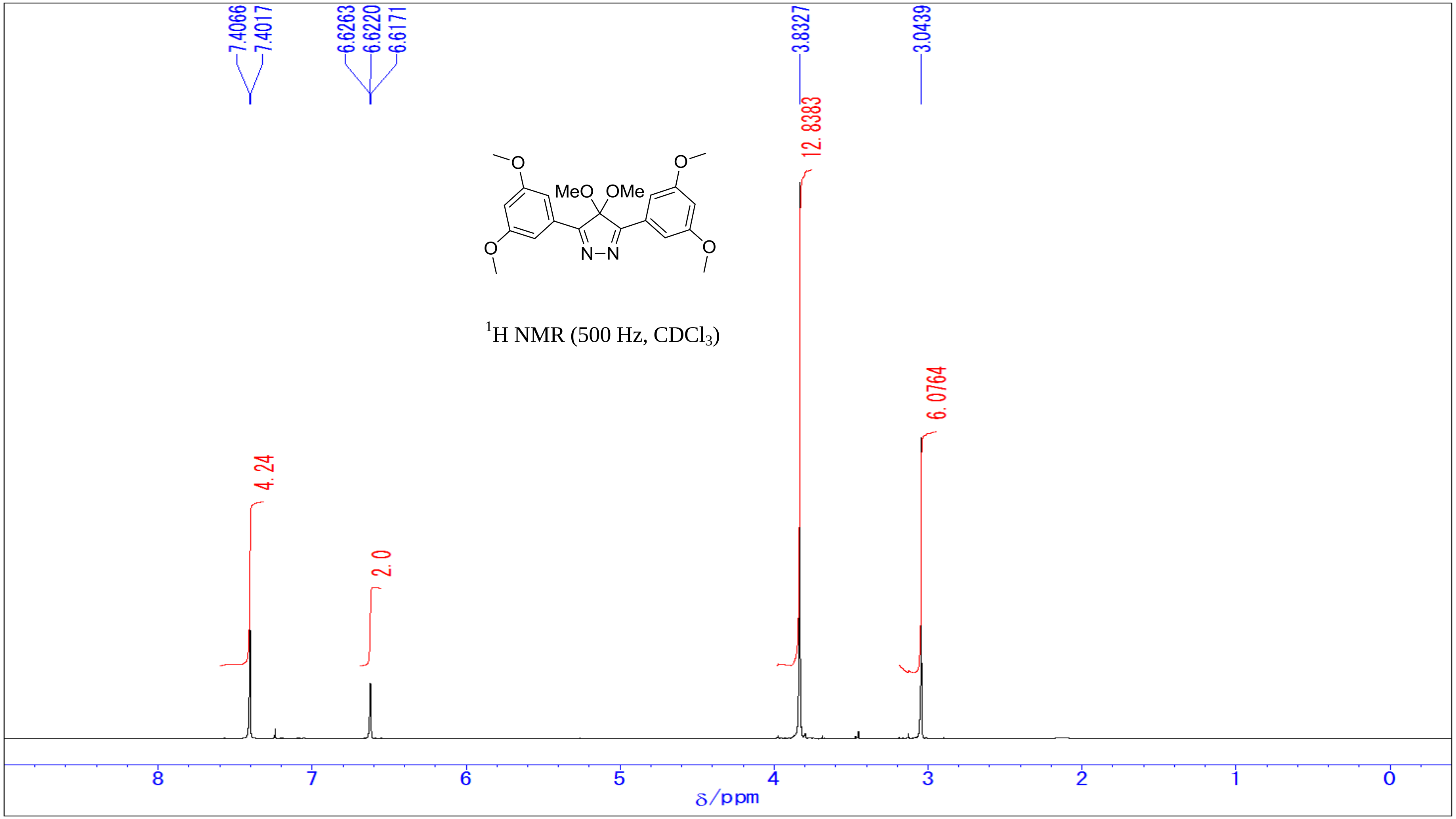

4,4-Dimethoxy-3,5-bis(3,5-dimethoxyphenyl)pyrazole (3g) ^{13}C NMR

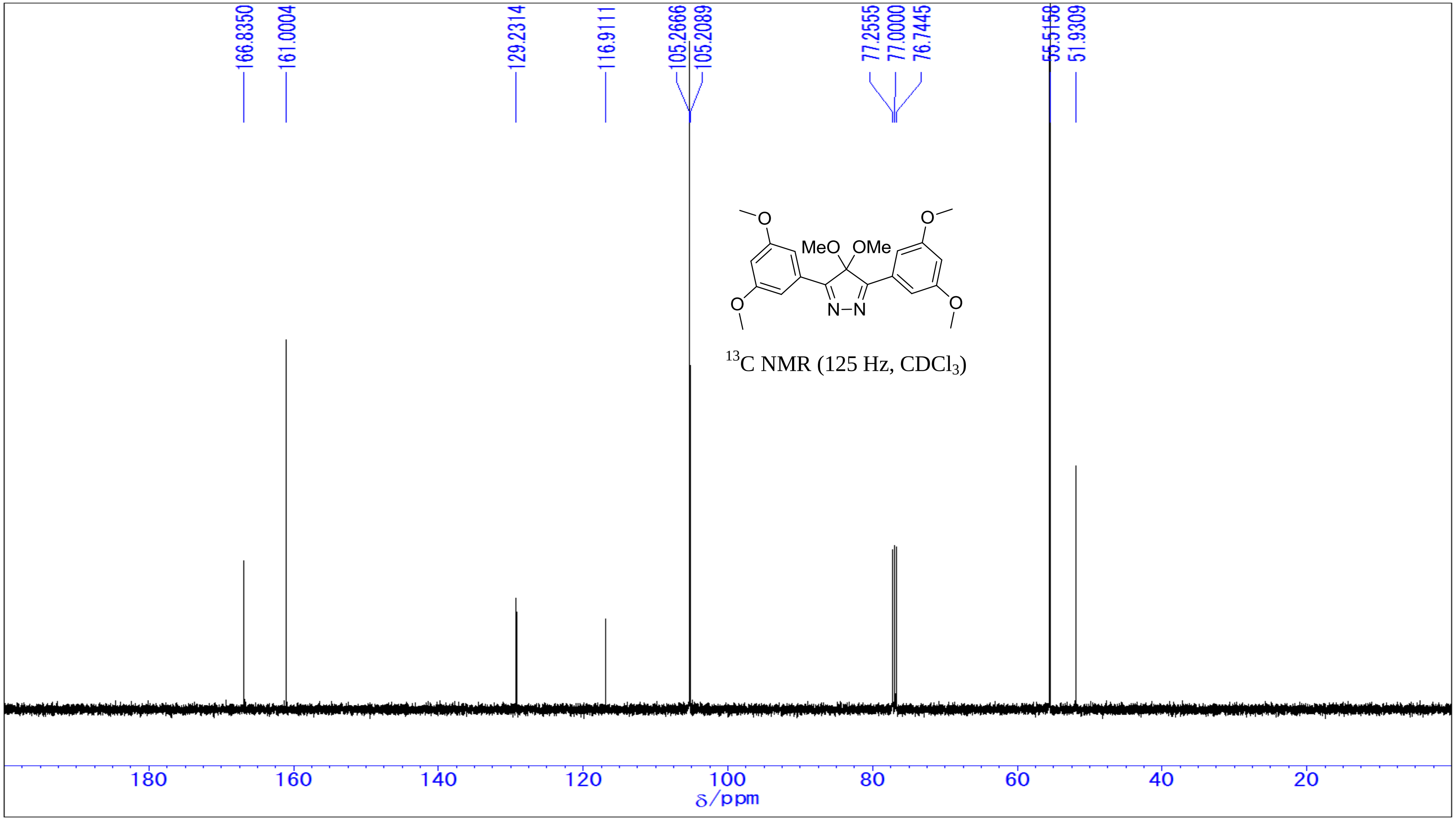

***endo*-2,3-Diazo-10,10-dibenzyloxy-1,4-diphenyltricyclo[5.2.1.0^{5,9}]dec-2-ene (AZc) ^{1}H NMR**

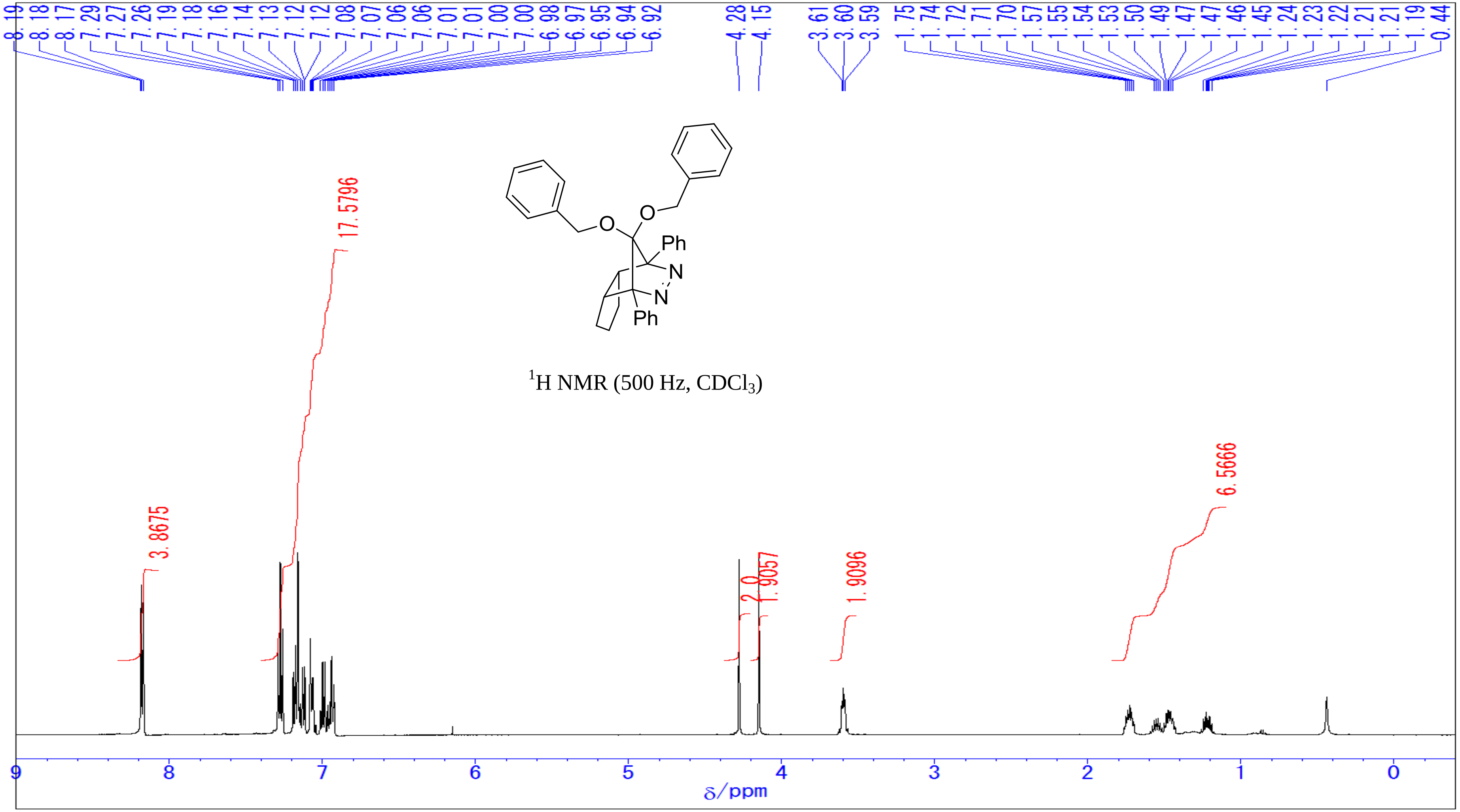

***endo*-2,3-Diazo-10,10-dibenzyloxy-1,4-diphenyltricyclo[5.2.1.0^{5,9}]dec-2-ene (AZc) ^{13}C NMR**

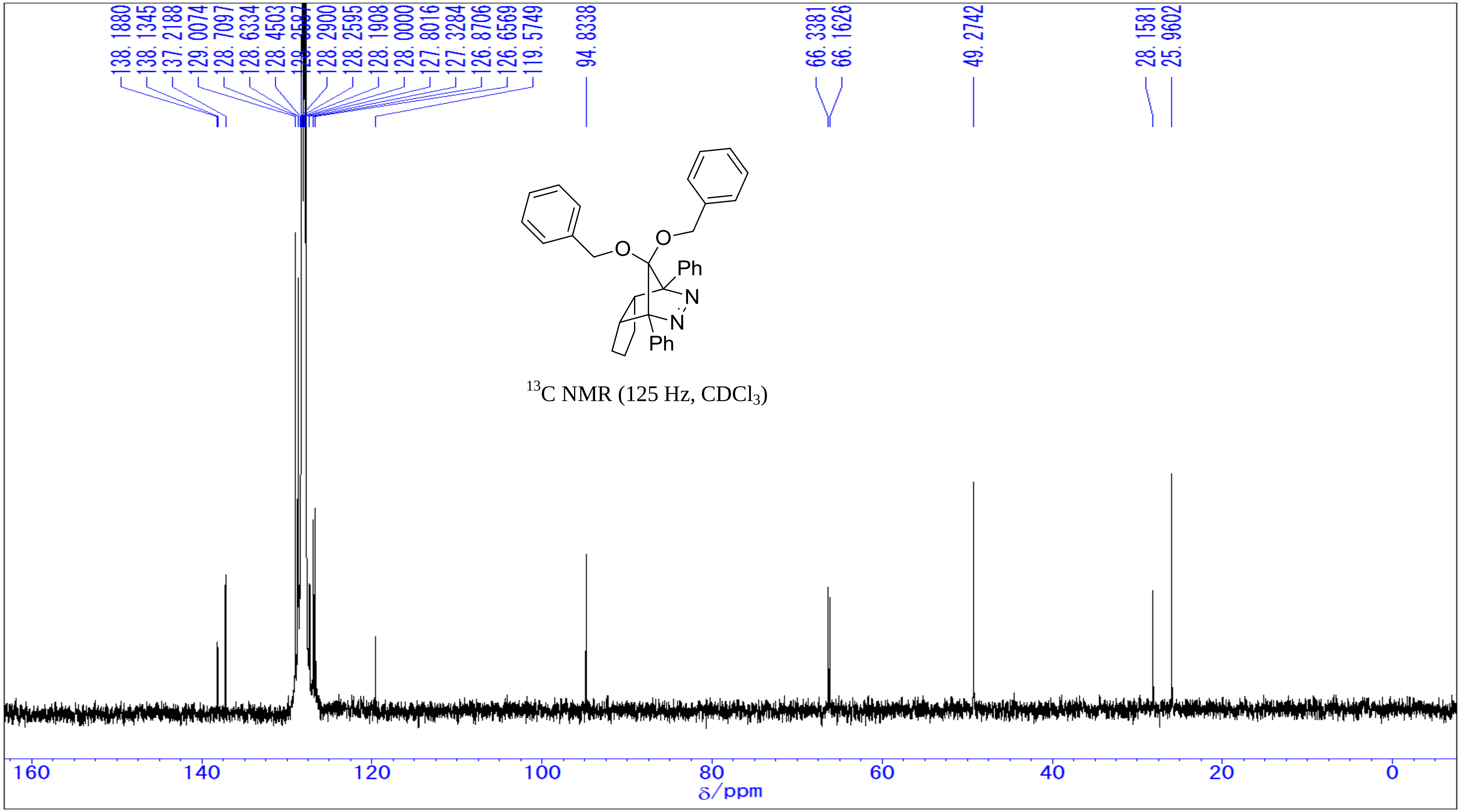

***endo*-2,3-Diazo-10,10-bis(3,5-dimethoxybenzyloxy)-1,4-diphenyltricyclo[5.2.1.0^{5,9}]dec-2-ene (AZd) ^{1}H NMR**

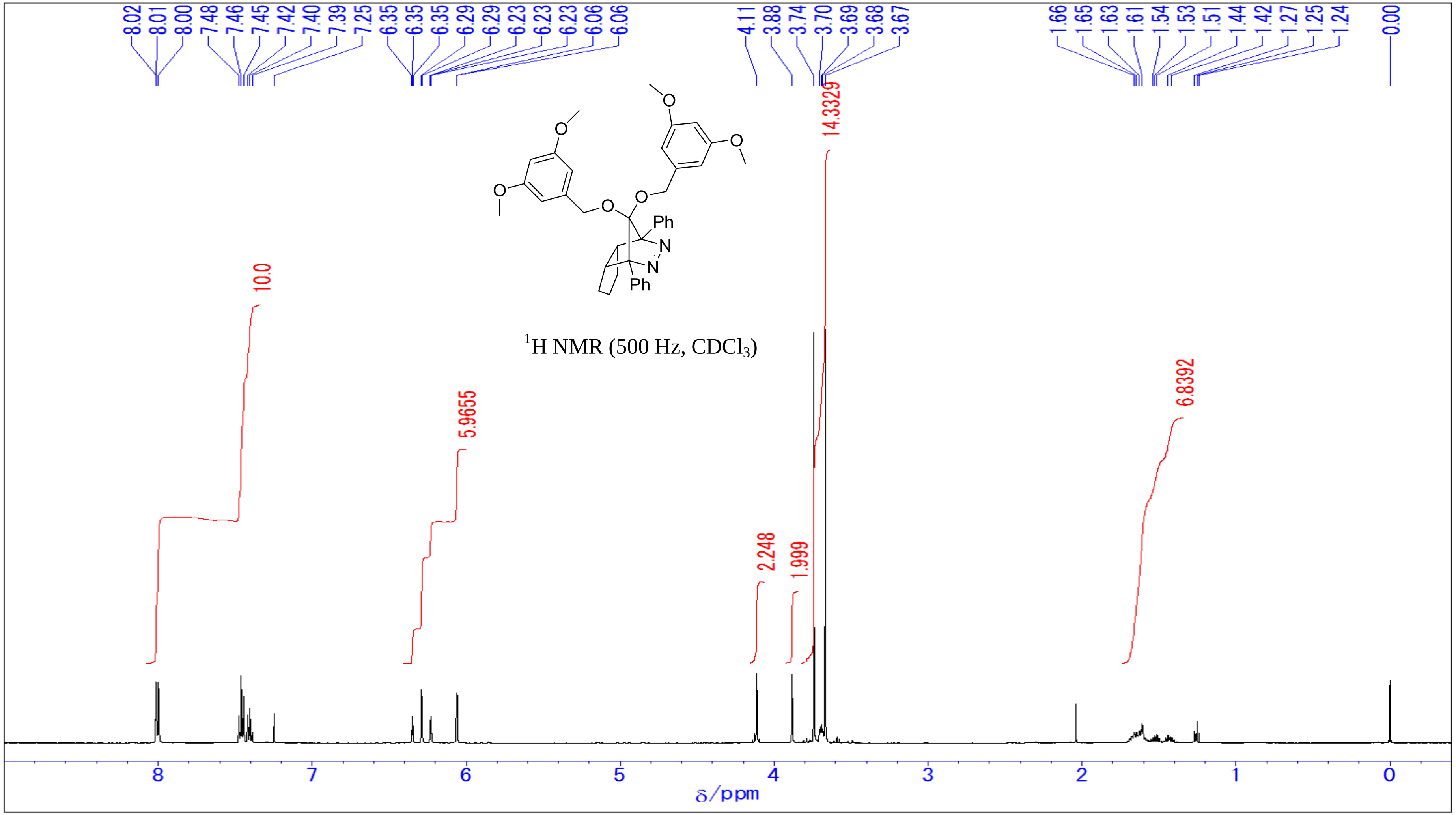

***endo*-2,3-Diazo-10,10-bis(3,5-dimethoxybenzyloxy)-1,4-diphenyltricyclo[5.2.1.0^{5,9}]dec-2-ene (AZd) ^{13}C NMR**

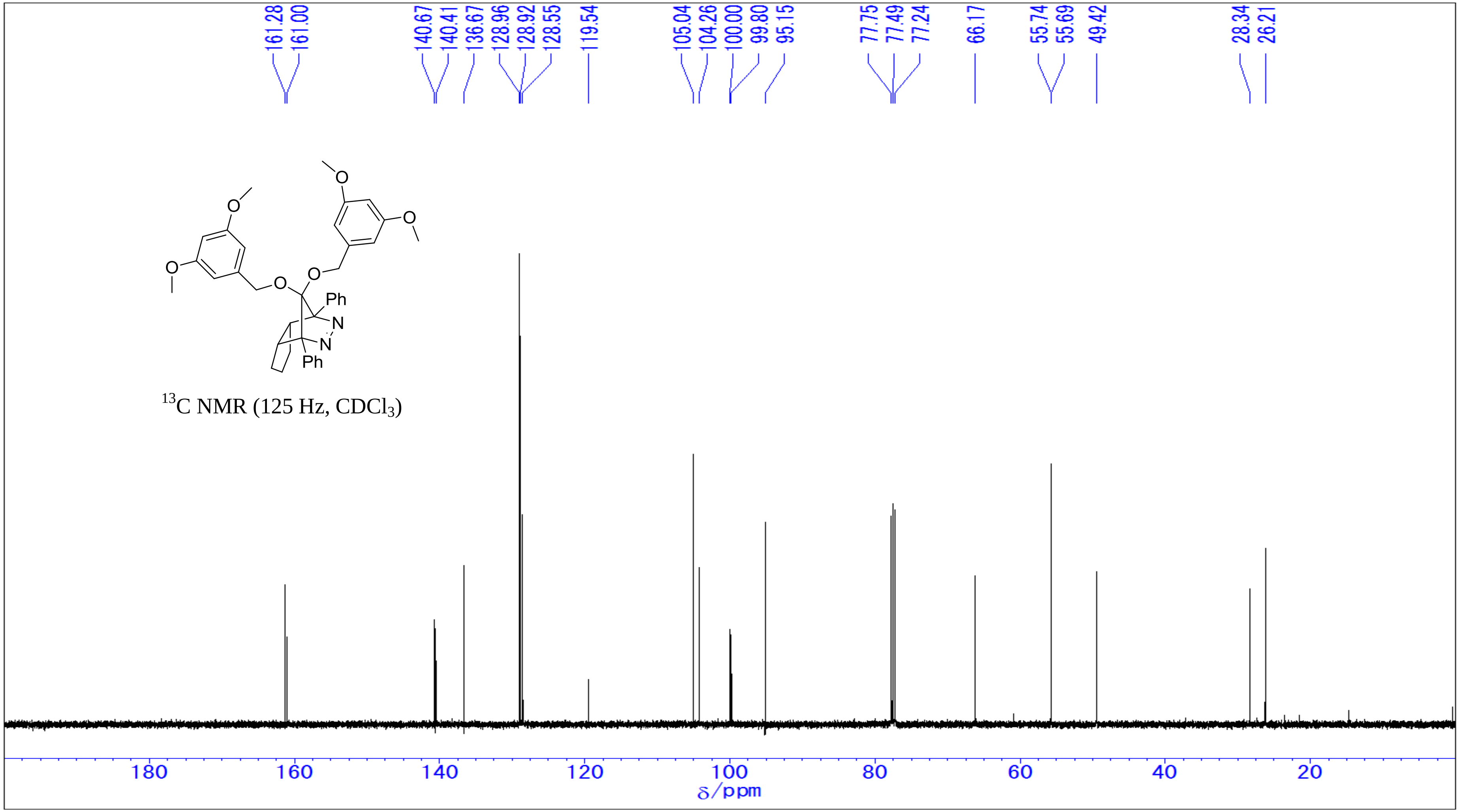

endo-2,3-Diazo-1,4-bis(3,5-dimethoxybenzyloxy)-10,10-dibenzyloxytricyclo[5.2.1.0^{5,9}]dec-2-ene (AZe) ^{1}H NMR

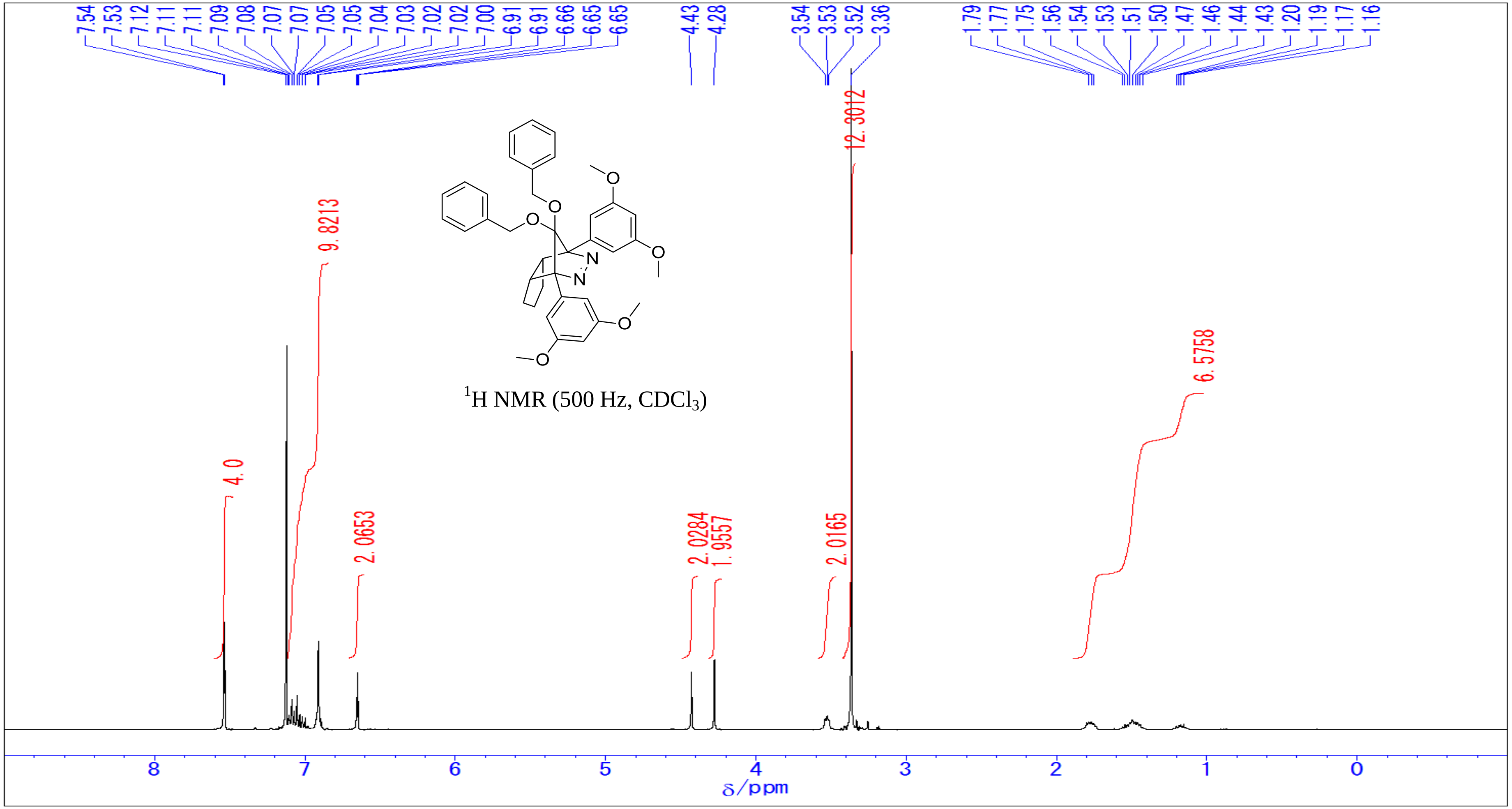

***endo*-2,3-Diazo-1,4-bis(3,5-dimethoxybenzyloxy)-10,10-dibenzyloxytricyclo[5.2.1.0^{5,9}]dec-2-ene (AZe) ^{13}C NMR**

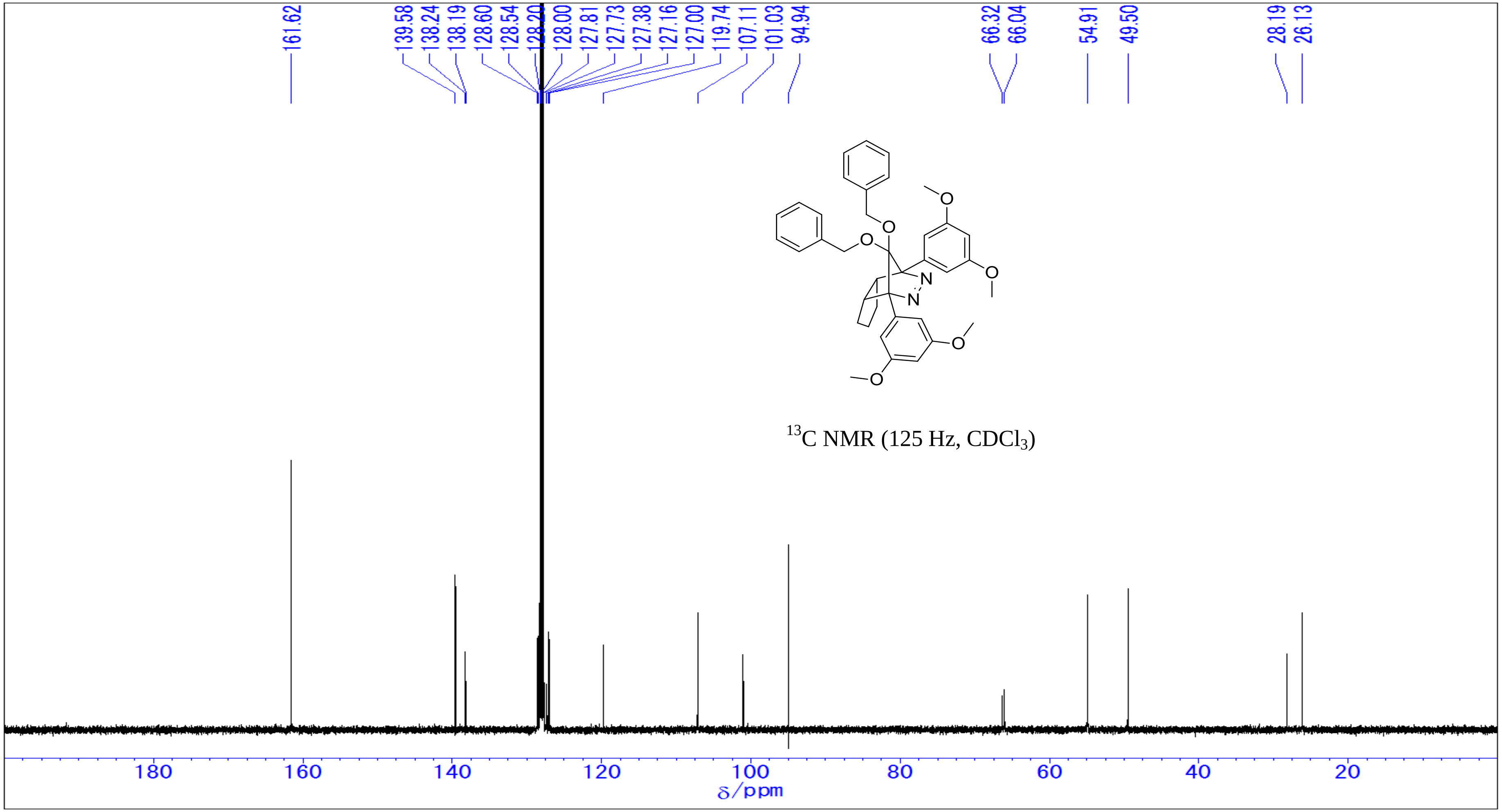

***endo*-2,3-Diazo-1,4-bis(3,5-dimethoxybenzyloxy)-10,10-bis(3,5-dimethoxyphenoxy)tricyclo[5.2.1.0^{5,9}]dec-2-ene (AZf) ^{1}H NMR**

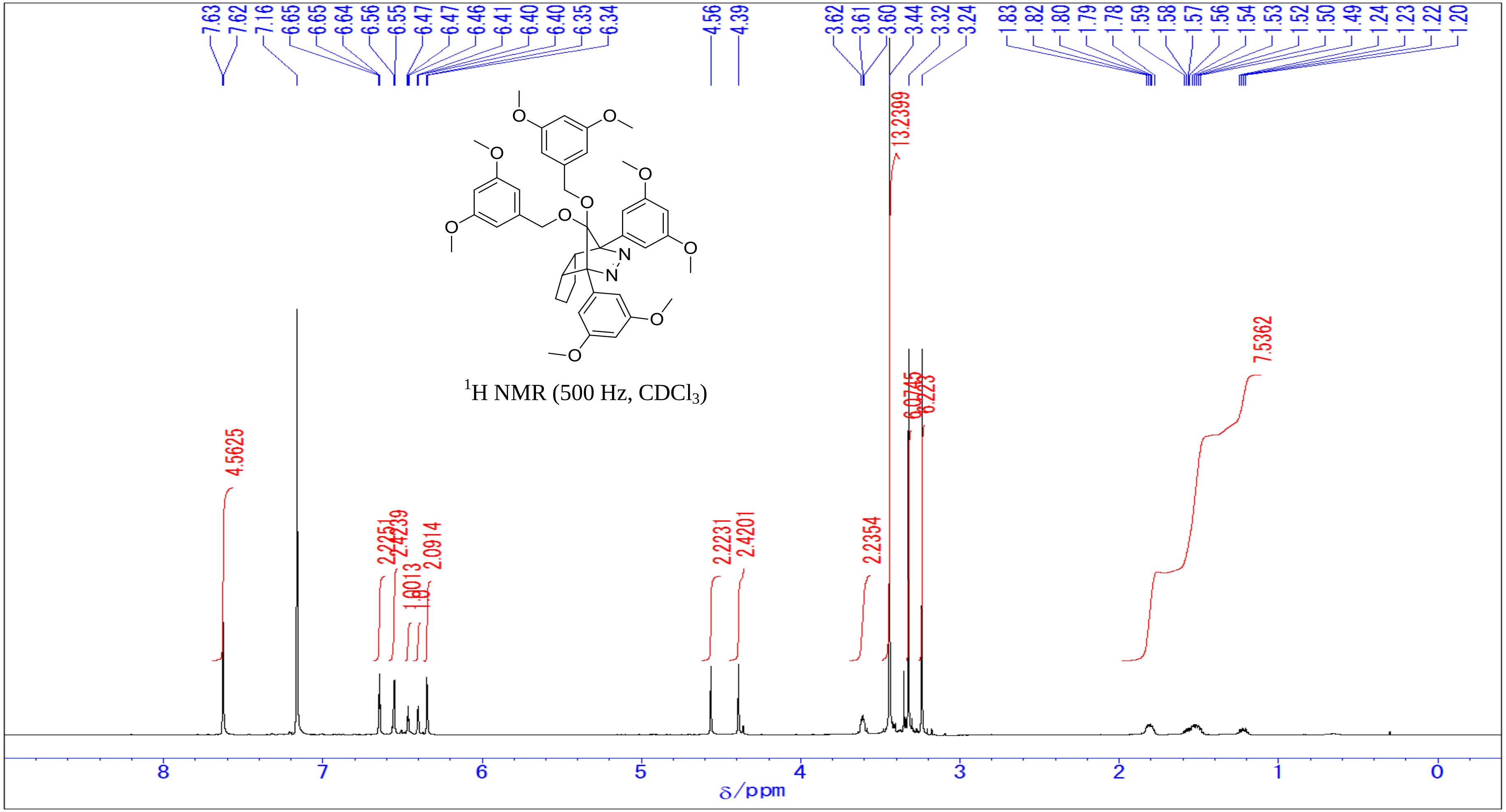

endo-2,3-Diazo-1,4-bis(3,5-dimethoxybenzyloxy)-10,10-bis(3,5-dimethoxyphenoxy)tricyclo[5.2.1.0^{5,9}]dec-2-ene (AZf) ^{13}C NMR

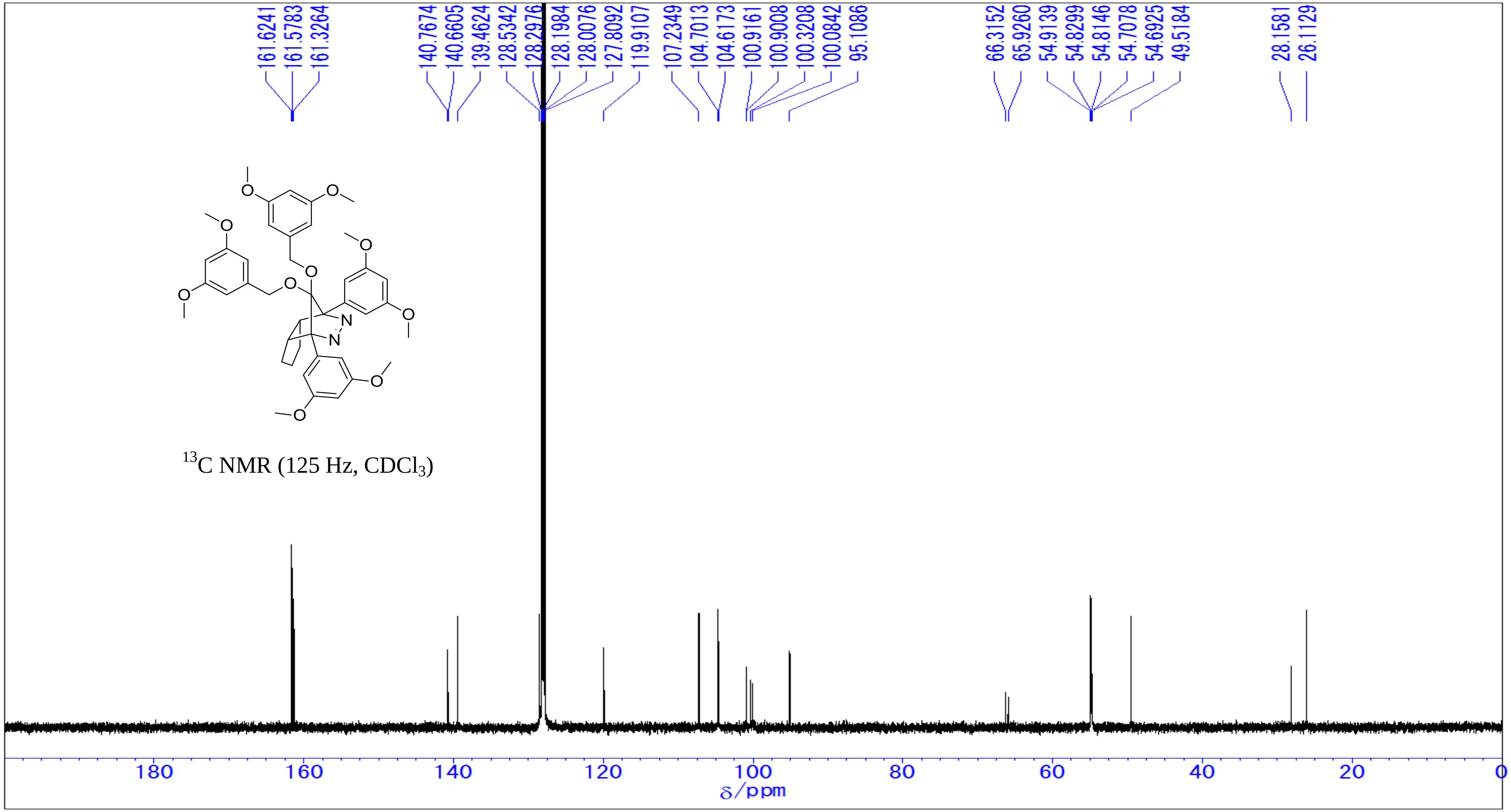

***endo*-2,3-Diazo-1,4-bis(3,5-dimethoxybenzyloxy)-10,10-dimethoxytricyclo[5.2.1.0^{5,9}]dec-2-ene (AZg) ^{1}H NMR**

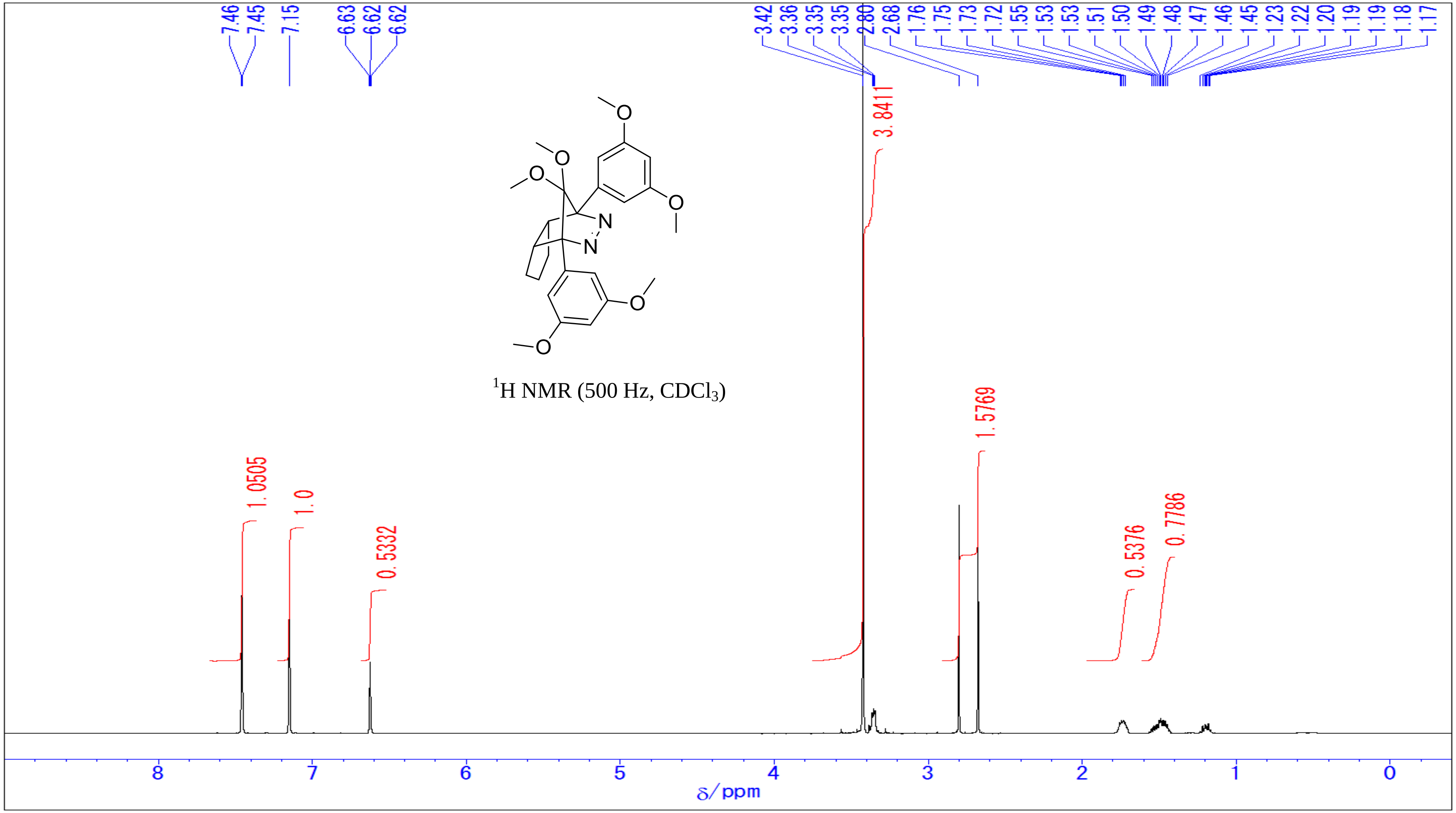

***endo*-2,3-Diazo-1,4-bis(3,5-dimethoxybenzyloxy)-10,10-dimethoxytricyclo[5.2.1.0^{5,9}]dec-2-ene (AZg) ^{13}C NMR**

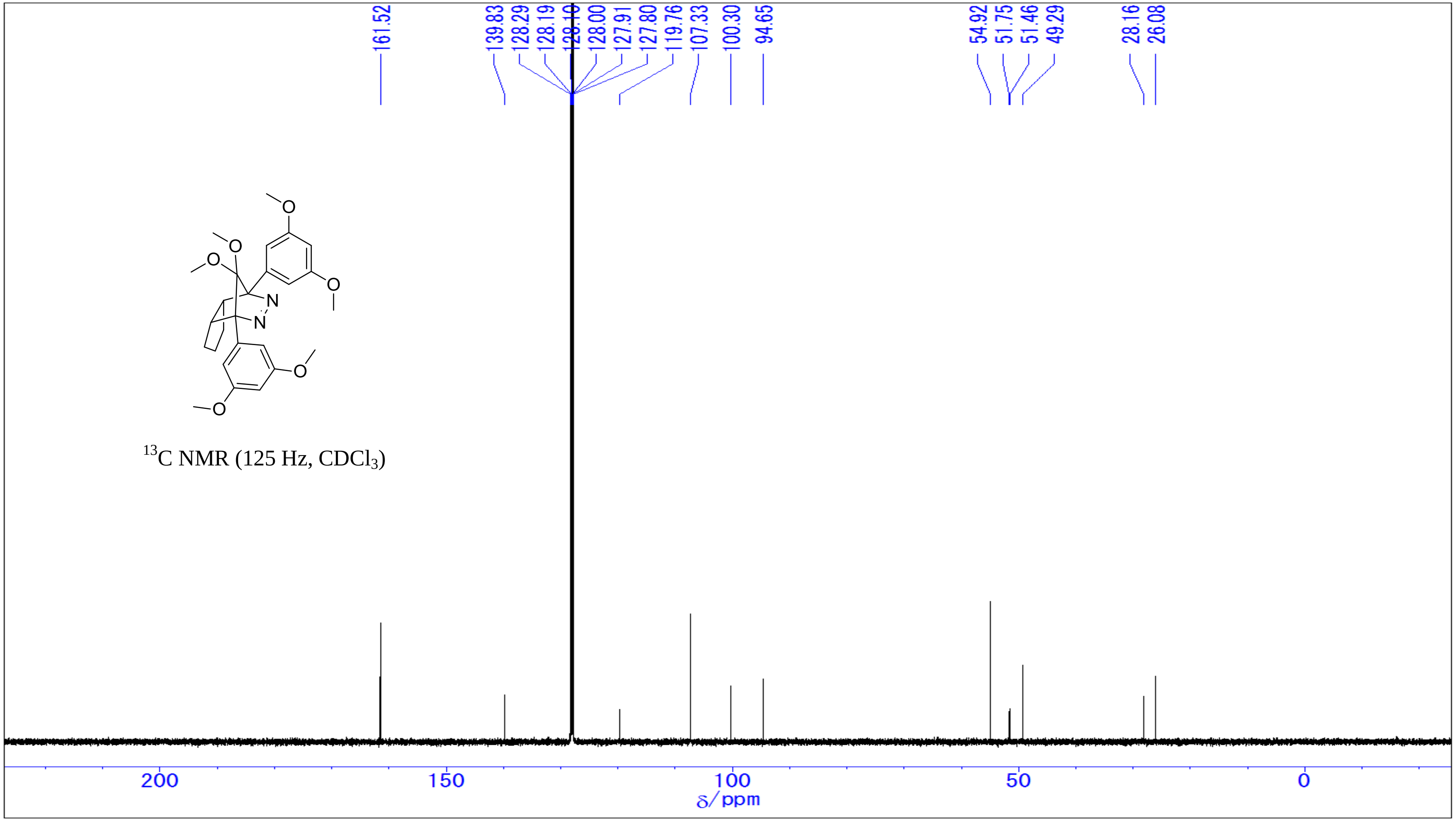

3,3-Dibenzyloxy-2,4-diphenyltricyclo[3.3.0.^{1,5}0^{2,4}]octane (CPc) ^{1}H NMR

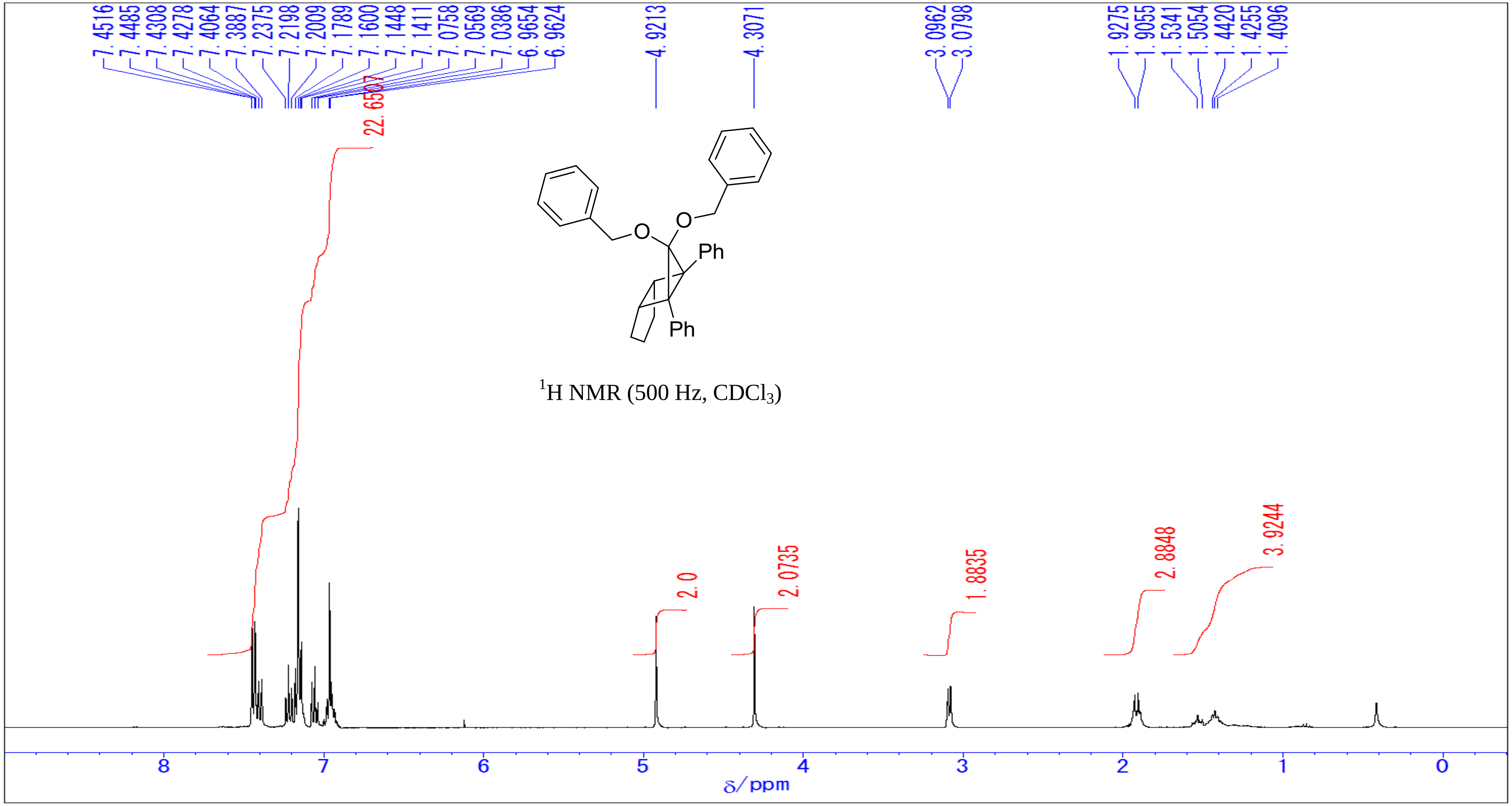

3,3-Dibenzyloxy-2,4-diphenyltricyclo[3.3.0.^{1,5}0^{2,4}]octane (CPc) ^{13}C NMR

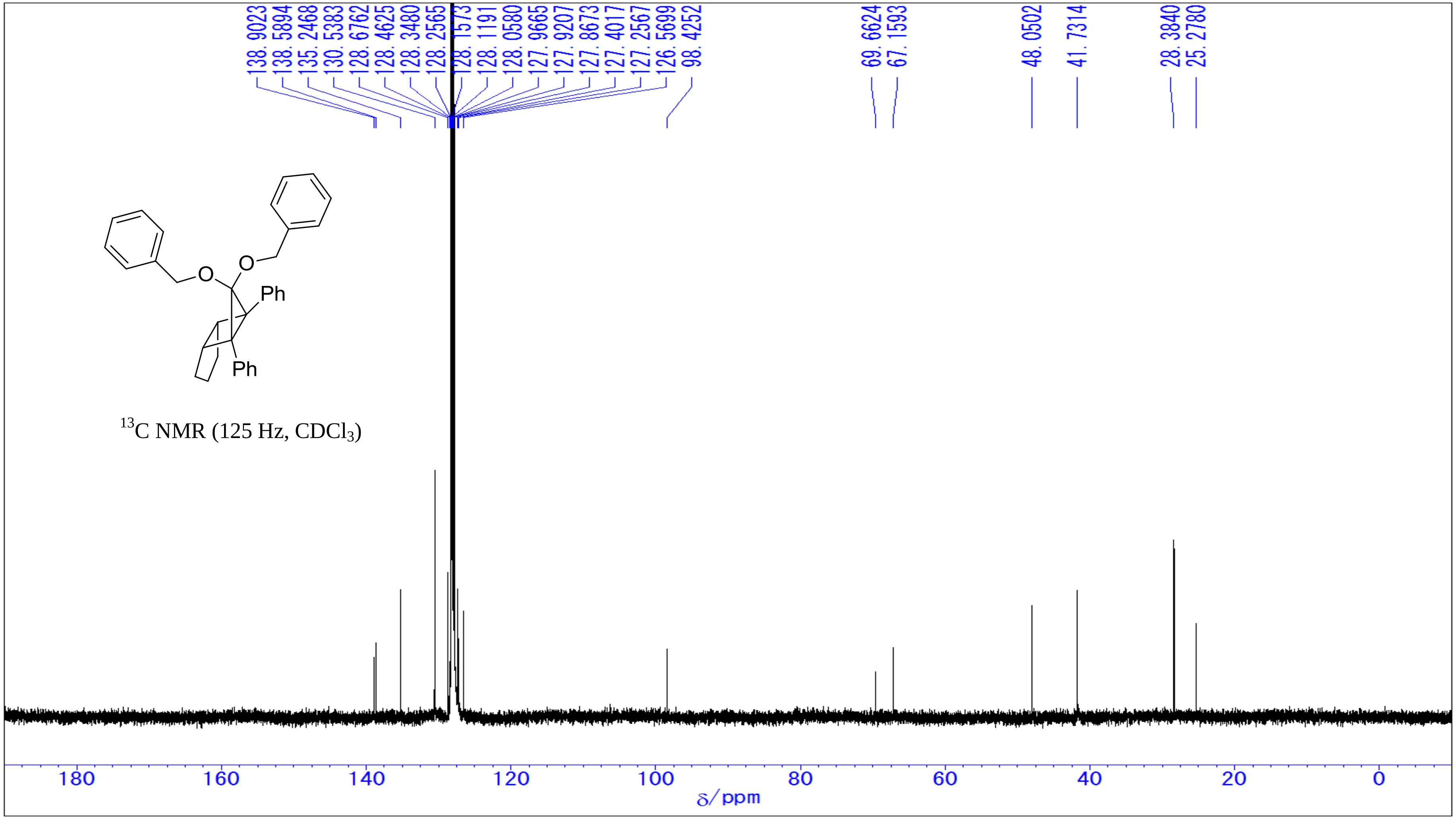

3,3-Bis(3,5-dimethoxybenzyloxy)-2,4-diphenyltricyclo[3.3.0.[1,5]0[2,4]]octane (CPd) ^{1}H NMR

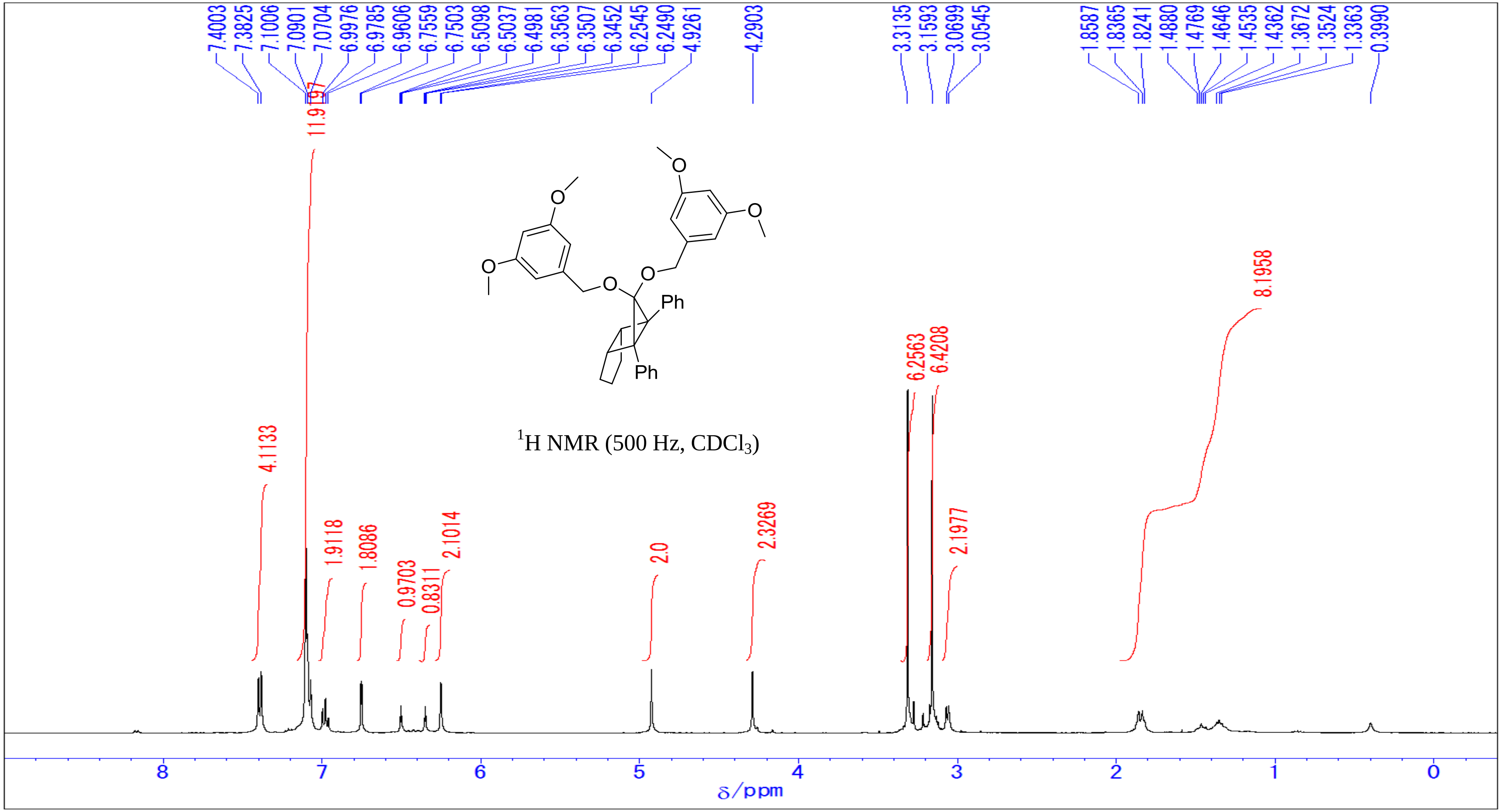

3,3-Bis(3,5-dimethoxybenzyloxy)-2,4-diphenyltricyclo[3.3.0.^{1,5}0^{2,4}]octane (CPd) ^{13}C NMR

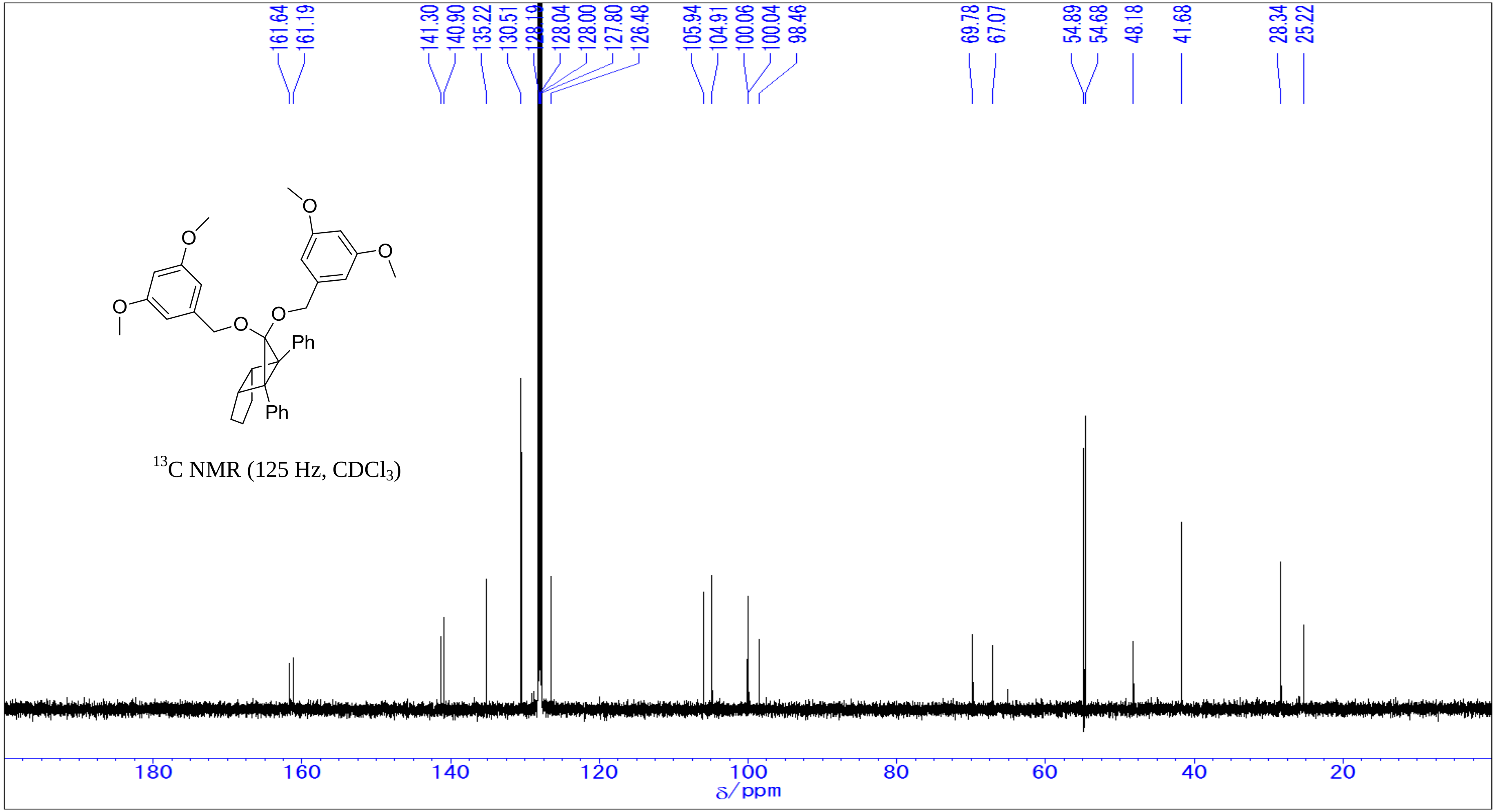

3,3-Dibenzyloxy-2,4-bis(3,5-dimethoxyphenyl)tricyclo[3.3.0.^{1,5}0^{2,4}]octane (CPe) ^{1}H NMR

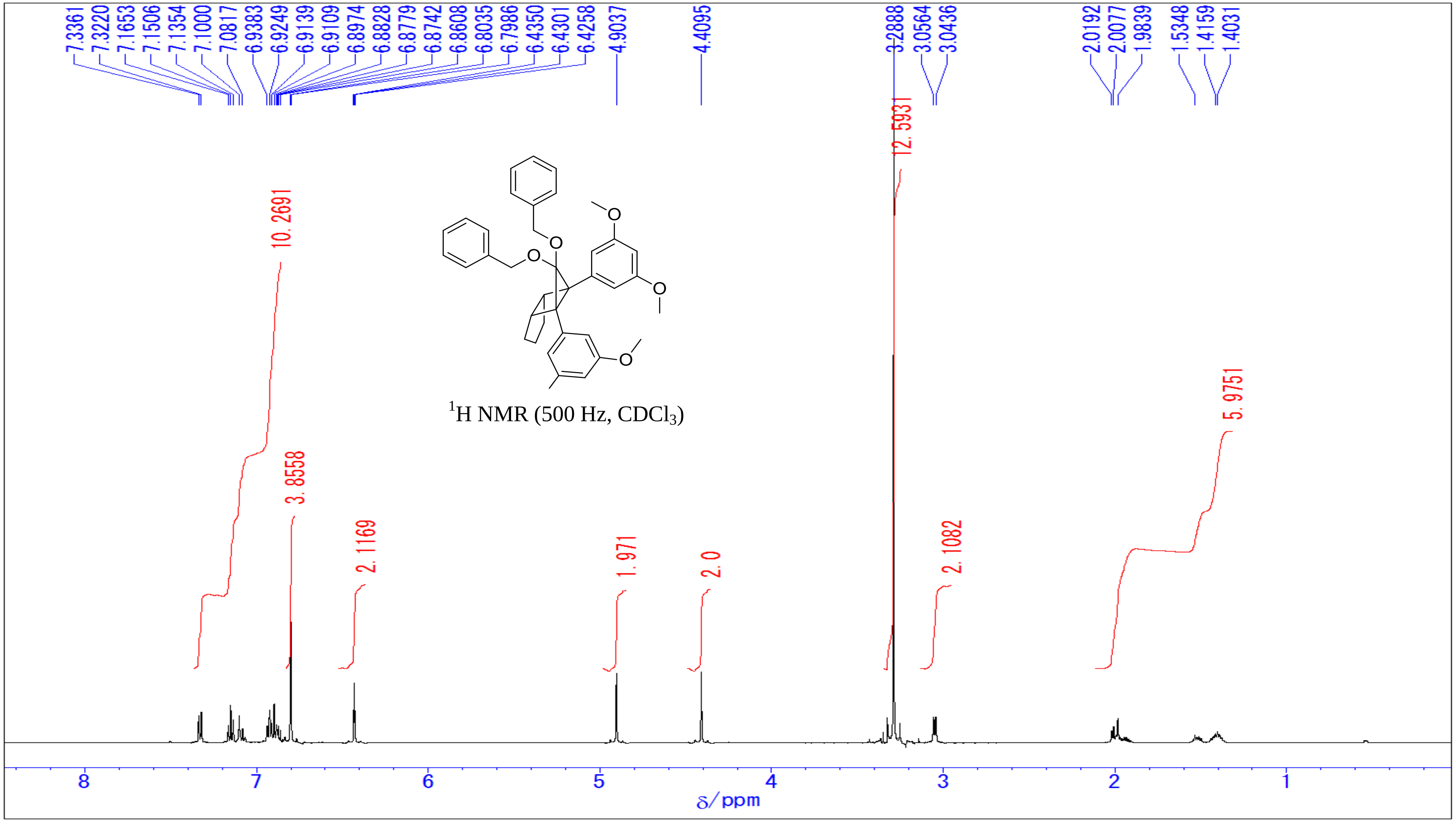

3,3-Dibenzyloxy-2,4-bis(3,5-dimethoxyphenyl)tricyclo[3.3.0.^{1,5}0^{2,4}]octane (CPe) ^{13}C NMR

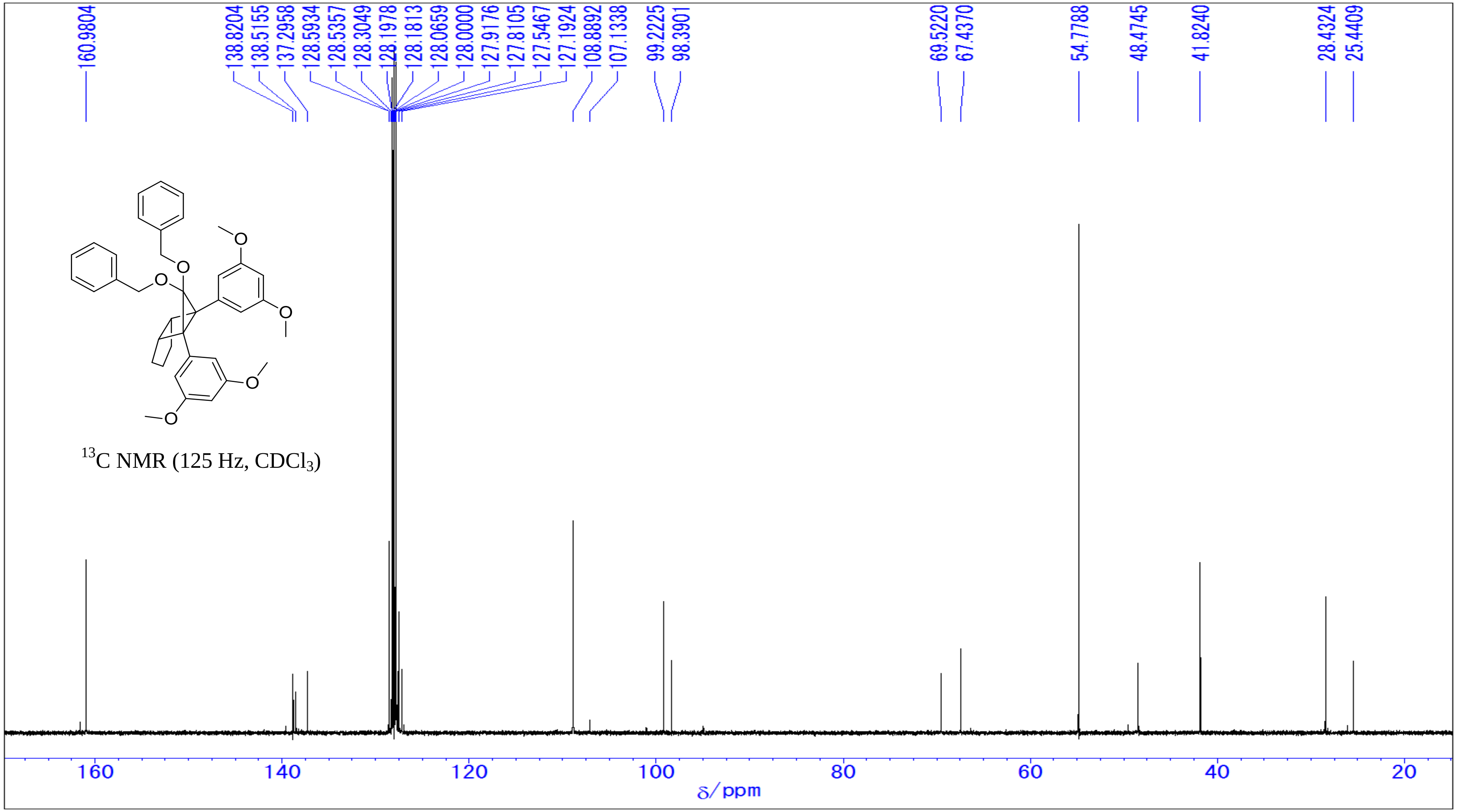

3,3-Bis(3,5-dimethoxybenzyloxy)-2,4-bis(3,5-dimethoxyphenyl)tricyclo[3.3.0.^{1,5}0^{2,4}]octane (CPf) ^{1}H NMR

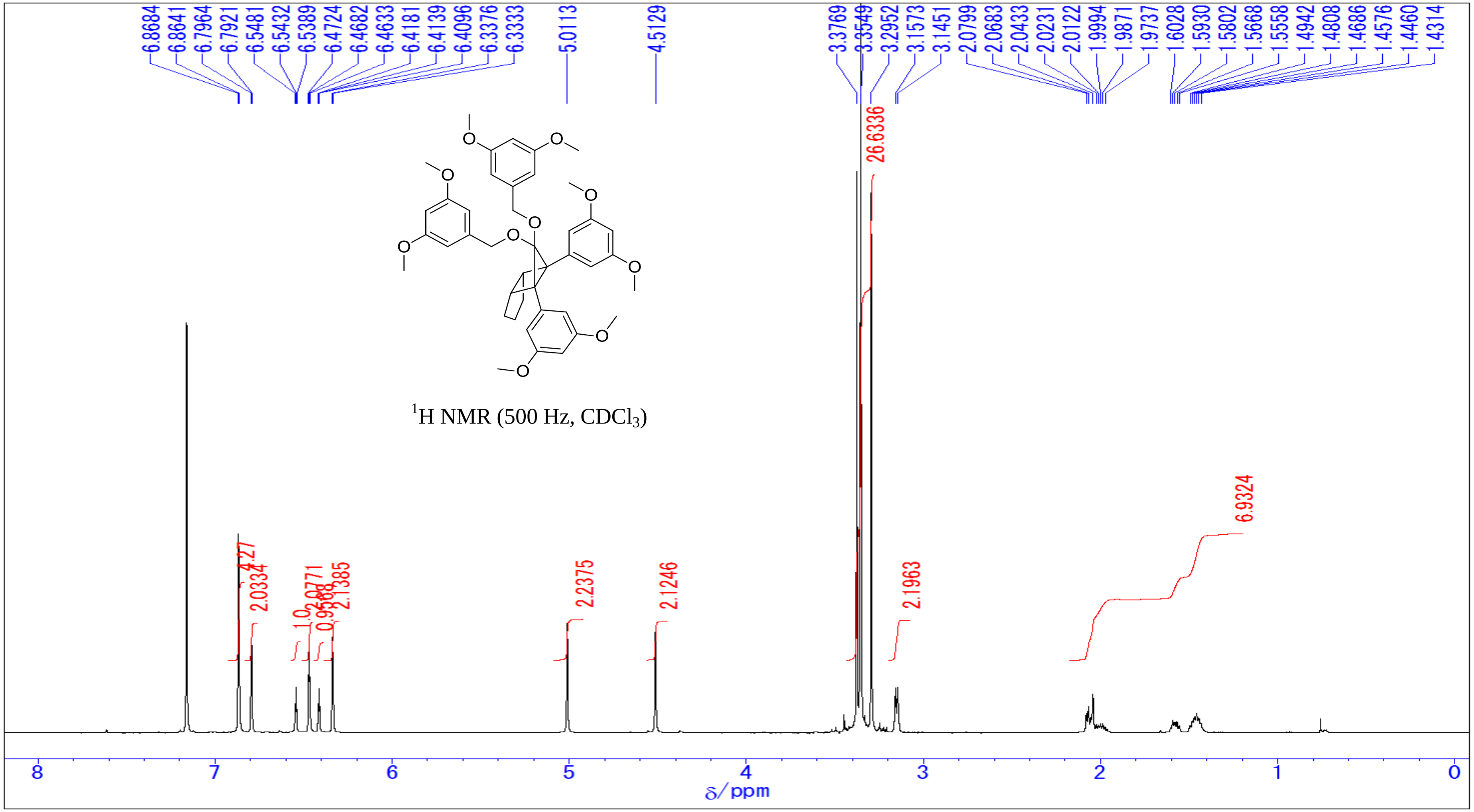

3,3-Bis(3,5-dimethoxybenzyloxy)-2,4-bis(3,5-dimethoxyphenyl)tricyclo[3.3.0.^{1,5}0^{2,4}]octane (CPf) ^{13}C NMR

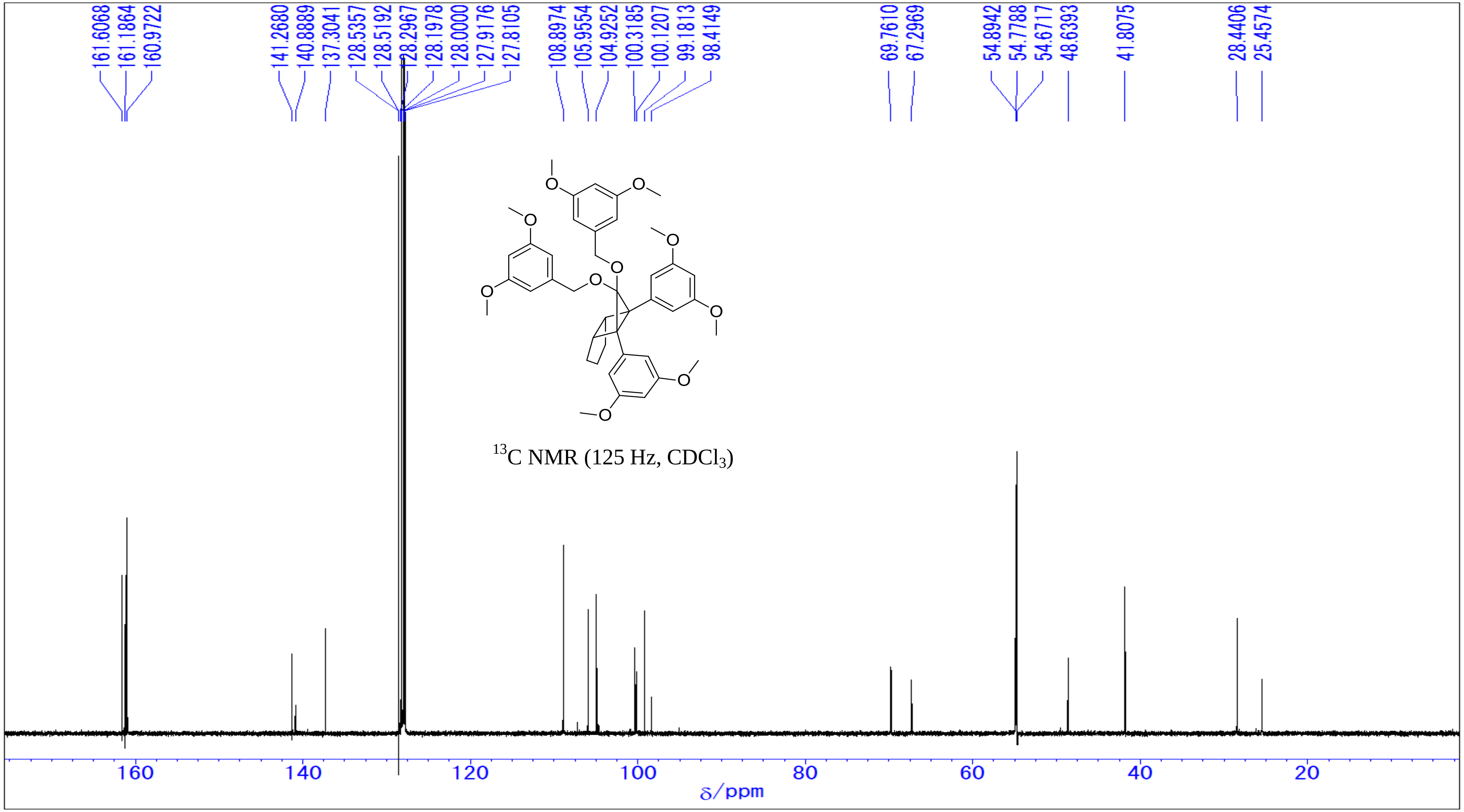

3,3-Dimethoxy-2,4-bis(3,5-dimethoxyphenyl)tricyclo[3.3.0.[1,5]0[2,4]]octane (CPg) ^{1}H NMR

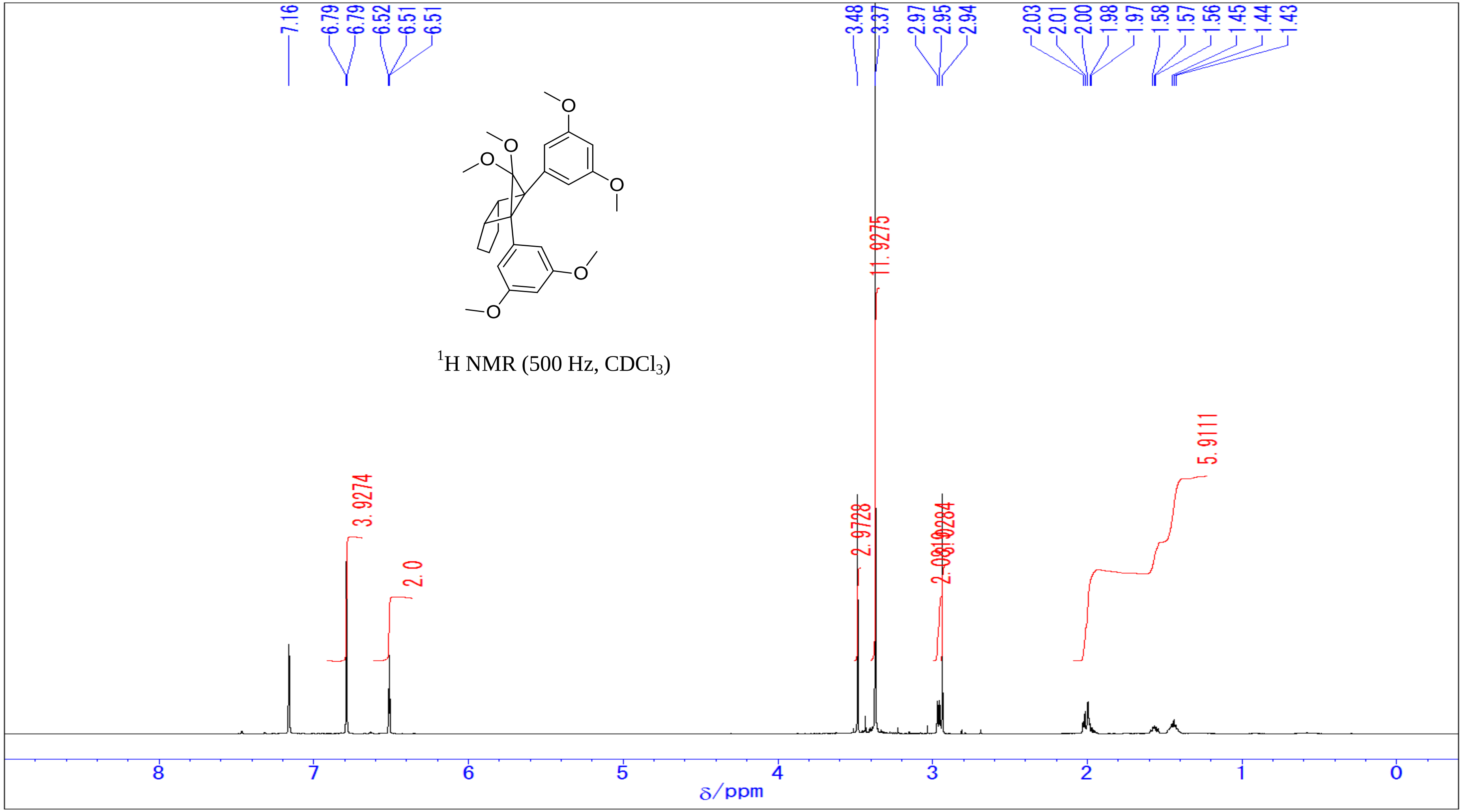

3,3-Dimethoxy-2,4-bis(3,5-dimethoxyphenyl)tricyclo[3.3.0.^{1,5}0^{2,4}]octane (CPg) ^{13}C NMR

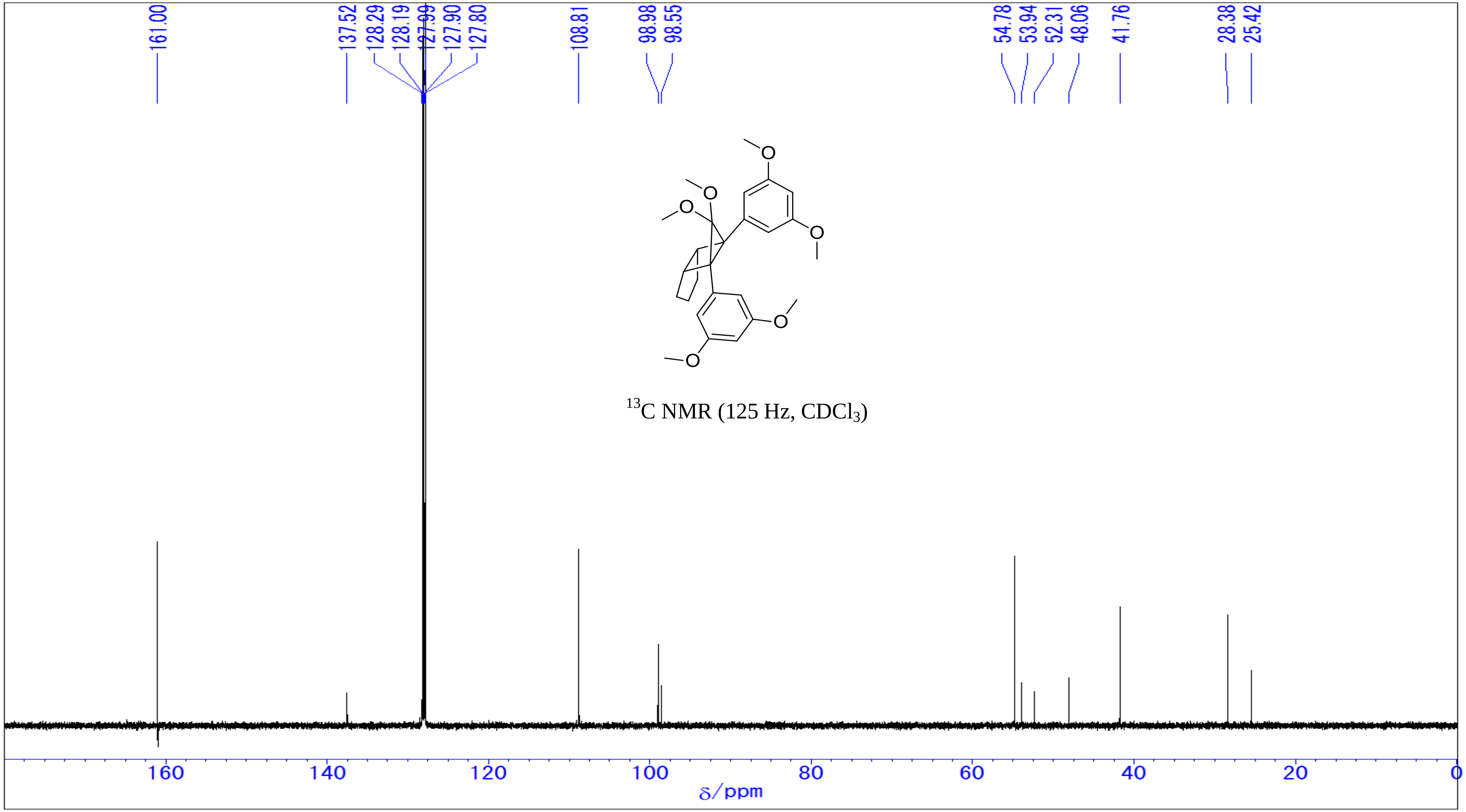

Supplement: File 1 — NMR spectra of compounds 1–5, AZc–g, and CPc–g. [file Beilstein_J_Org_Chem-09-925-s001.pdf]
